# Prediction models for postoperative pulmonary complications in intensive care unit patients after noncardiac thoracic surgery

**DOI:** 10.1186/s12890-024-03153-z

**Published:** 2024-08-29

**Authors:** Xiangjun He, Meiling Dong, Huaiyu Xiong, Yukun Zhu, Feng Ping, Bo Wang, Yan Kang

**Affiliations:** Department of Critical Care Medicine, West China Hospital, Sichuan University and Institute of Critical Care Medicine, No. 17, Section 3, Renmin South Road, Wuhou District, Chengdu City, Sichuan Province 610041 China

**Keywords:** Postoperative pulmonary complications, Intensive care unit, Noncardiac thoracic surgery, Risk factor, Nomogram

## Abstract

**Background:**

Postoperative pulmonary complication (PPC) is a leading cause of mortality and poor outcomes in postoperative patients. No studies have enrolled intensive care unit (ICU) patients after noncardiac thoracic surgery, and effective prediction models for PPC have not been developed. This study aimed to explore the incidence and risk factors and construct prediction models for PPC in these patients.

**Methods:**

This study retrospectively recruited patients admitted to the ICU after noncardiac thoracic surgery at West China Hospital, Sichuan University, from July 2019 to December 2022. The patients were randomly divided into a development cohort and a validation cohort at a 70% versus 30% ratio. The preoperative, intraoperative and postoperative variables during the ICU stay were compared. Univariate and multivariate logistic regression analyses were applied to identify candidate predictors, establish prediction models, and compare the accuracy of the models with that of reported risk models.

**Results:**

A total of 475 ICU patients were enrolled after noncardiac thoracic surgery (median age, 58; 72% male). At least one PPC occurred in 171 patients (36.0%), and the most common PPC was pneumonia (153/475, 32.21%). PPC significantly increased the duration of mechanical ventilation (*p* < 0.001), length of ICU stay (*p* < 0.001), length of hospital stay (LOS) (*p* < 0.001), and rate of reintubation (*p* = 0.047) in ICU patients. Seven risk factors were identified, and then the prediction nomograms for PPC were constructed. At ICU admission, the area under the curve (AUC) was 0.766, with a sensitivity of 0.71 and specificity of 0.60; after extubation, the AUC was 0.841, with a sensitivity of 0.75 and specificity of 0.83. The models showed robust discrimination in both the development cohort and the validation cohort, and they were well calibrated and more accurate than reported risk models.

**Conclusions:**

ICU patients who underwent noncardiac thoracic surgery were at high risk of developing PPCs. Prediction nomograms were constructed and they were more accurate than reported risk models, with excellent sensitivity and specificity. Moreover, these findings could help assess individual PPC risk and enhance postoperative management of patients.

**Supplementary Information:**

The online version contains supplementary material available at 10.1186/s12890-024-03153-z.

## Background

Postoperative pulmonary complications (PPC) including respiratory tract infection, pleural effusion, respiratory failure, and acute respiratory distress syndrome (ARDS), are commonly observed in postoperative patients [1–3]. The impact of PPC varies, leading to postoperative morbidity, intensive care unit (ICU) admission, in-hospital mortality, and prolonged length of hospital stay(LOS) [2, 4, 5]. However, previous investigators have primarily concentrated on specific subgroups of postoperative patients, such as those undergoing lobectomy, hepatectomy, abdominal surgery, or cardiac surgery [6–13]. Studies that specifically investigate the occurrence of PPCs in patients undergoing general thoracic surgeries are rare. Additionally, while previous studies have acknowledged ICU admission as a postoperative complication, reports of the incidence of PPCs in ICU patients are rare [4, 14]. The PPC studies enrolled ICU patients who underwent hepatectomy, on-pump cardiac surgery, or noncardiothoracic surgery [8, 11, 14]. However, ICU patients who underwent noncardiac thoracic surgery have not been included.

The prediction of PPCs could enhance the provision of personalized care for patients and contribute to the efficient allocation of limited resources. Prior research has identified various risk factors for PPC, including sex, age, body mass index (BMI), smoking status, chronic obstructive pulmonary disease (COPD), forced expiratory volume in one second (FEV1), and intraoperative variables [1, 6, 7, 15–18]. The prevalence and screening of lung cancer and esophageal tumors have significantly increased, leading to an increase in the number of thoracic surgeries performed globally [19–21]. With the increasing prevalence of video-assisted thoracic surgery(VATS) and robot-assisted thoracic surgery(RATS) [22, 23], different risk factors are emerging and prediction models for PPCs need to be updated. Several studies have proposed prediction models for PPC [1, 12, 15, 16]. Only the association between the American Society of Anesthesiologists (ASA) grade and the Assess Respiratory Risk in Surgical Patients in Catalonia (ARISCAT score) and the occurrence of PPC was confirmed [1, 24, 25]. Several risk models of postoperative morbidity in patients undergoing thoracic surgery from American or European Thoracic Surgeons database have been reported [26–28], but they have been used for specific types of patients and have not been validated in ICU patients. There is a lack of prediction models for PPC in ICU patients who underwent noncardiac thoracic surgery. Therefore, this study aimed to examine the occurrence of PPC events in postoperative ICU patients who underwent noncardiac thoracic surgery, explore the risk factors and establish new prediction models for PPCs.

## Methods

### Ethics and study design

The ethical committee of West China Hospital, Sichuan University approved this retrospective study (20,221,074). Informed consent was waived due to the retrospective and noninterventional design. All the authors followed the Declaration of Helsinki.

This study was conducted in the Department of Critical Care Medicine, West China Hospital, Sichuan University. This study aimed to investigate the incidence of PPC events in ICU patients after noncardiac thoracic surgery; identify risk factors for postoperative pulmonary complications, develop prediction models for PPC; assess and compare the predictive value of established models for PPC with reported risk models. Besides, we aimed to construct staged prediction models based on the timeline of patients after admitted to the ICU, one at ICU admission and one after extubation. At ICU admission, the risk for PPCs was assessed, to identify high-risk patients and improve management. After extubation, those patients reevaluated at high risk of PPCs could be intervened earlier.

All patients admitted to the ICU from July 2019 to December 2021 at West China Hospital, Sichuan University were enrolled. Patients who meet one of the following criteria were considered to transfer to the ICU: (1) Older than 75 years old; (2) With difficulty in extubation after surgery; (3) Required vasoactive agents to stabilize hemodynamics; (4) At high risk of postoperative complications considered by surgeon or anaesthesiologist. Patients were included if they were: (1) at least 18 years old; (2) admitted to the ICU after surgery (including emergency surgery, limited surgery, or elective surgery); (3) underwent surgery including lung, mediastinum, esophagus or thoracic wall; (4) intubated at ICU admission. Patients were excluded from enrollment if they were: (1) scheduled for pregnancy-related surgery; (2) or not immediately admitted to the ICU after surgery; (3) or secondarily admitted to ICU; (4) or whose length of ICU stay was less than 24 h. Perioperative and postoperative care, including anaesthesia and analgesia protocols, fluid management, transfusion, nutritional support or ventilation strategy was performed at the discretion of the physician in charge. Extubation was performed after discussion among the surgeon, physician and respiratory therapist in charge. Spontaneous breathing trials were performed following the protocol of the American Association for Respiratory Care.

The sample size of this study was calculated following formulas reported [29]. A pilot study was performed before developing prediction models for PPC, and the incidence of PPC was 30%. The calculated sample size of the developing cohort was at least 332(7 predictors [1]), and the total was 474(7:3 ratio).

### Data collection and outcomes

Perioperative clinical data were collected following the predesigned collection forms (Supplemental 1). Preoperative data included patient demographics, medical history, pulmonary function test results including postoperative predictive FEV1 (FEV1-ppo, L) 30, baseline laboratory test results, ASA class, the ARISCAT score, the Eurolung1(2016) and Eurolung1(2019) scores [26, 27]. Intraoperative information included the surgical method, duration of surgery, prophylactic antibiotics, and liquid volume(mL). During the postoperative phase (in the ICU), the Acute Physiology and Chronic Health Evaluation II (APACHE II) score, vital signs, laboratory test results, imaging results, and ventilation strategies at ICU admission and after extubation were recorded during the seven postoperative days (pod).

The primary outcome was PPC events occurring from the first postoperative day (pod 0) until the seventh postoperative day (pod 7) or hospital discharge. The PPC was defined as a composite of the following: pneumonia, respiratory failure, pleural effusion (moderate to severe), acute respiratory distress syndrome (ARDS), pulmonary embolism, pneumothorax(moderate to severe), or bronchospasm [1, 3]. The presence and type of PPC were independently evaluated by two clinical physicians. The time of occurrence and the number of PPCs were also documented. Secondary outcomes included the length of ICU stay (d), length of hospital stay (LOS, d), duration of mechanical ventilation (duration of MV, h), events of re-intubation, ICU re-admission, in-hospital mortality, and automatic discharge, and other clinical outcomes.

### Statistical analysis

**Fig. 1:**
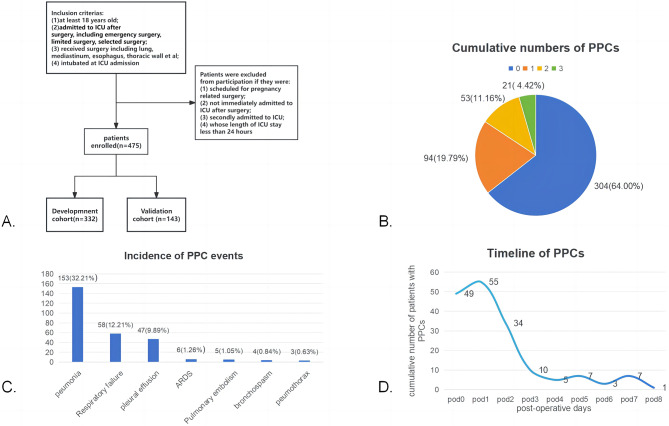
(**A**) Flow chart of the analysis. All patients underwent noncardiac thoracic surgery and fulfilled the inclusion criteria were enrolled, and patients were excluded if they fulfilled the exclusion criteria. Patients were subsequently divided into a development cohort and a validation cohort (7:3); (**B**) Cumulative number and percentage of patients with numerous PPCs; (**C**) Incidence of postoperative pulmonary complication events; (**D**) Timeline of occurrence of PPCs during the ICU stay Abbreviations: ARDS: acute respiratory distress syndrome; ICU: intensive care unit; pod: postoperative day; PPC: postoperative pulmonary complication

All the patients were divided into two groups by PPC events. Patients were categorized into several subgroups according to the specific type, cumulative number and time of occurrence of PPCs. Additionally, patients underwent different surgical procedures were divided into several groups. To develop prediction models, all the patients enrolled were randomly divided into a development cohort(*n* = 332) and a validation cohort (*n* = 143). The baseline characteristics of the two cohorts were also compared (Fig. 1A). Kolmogorov–Smirnov–Lilliefors test was used to test the normality of all the variables. Normally distributed continuous variables are represented by the mean and standard deviation (SD), and continuous variables with a skewed distribution are represented by the median and interquartile range (IQR). Categorical variables are represented by counts and percentages. Differences in baseline characteristics and outcomes are calculated by chi-square test, Fisher test or *Kruskal-Wallis* test for categorical variables, and t test or Wilcoxon rank sum test for continuous variables. A *p*-value less than 0.05 was considered statistically significant.

The association between each variable and PPC was examined by univariate logistic regression; a *p*-value less than 0.1 was considered a candidate predictor for PPC. The results are expressed as odds ratios (ORs) and 95% confidence intervals (CIs). Multivariate logistic regression analysis (stepwise, backwards elimination) [31] was subsequently used to determine the independent risk factors, and the results are expressed as OR values and 95% CIs; a *p*-value less than 0.05 was considered statistically significant. Then, nomograms for predicting the probability of PPC were established and receiver operating characteristic (ROC) curves were drawn with STATA V.16.0 software. We constructed staged prediction models based on the timeline of patients after admitted to the ICU, one at ICU admission and one after extubation, to compare the area under the curve (AUC), sensitivity, and specificity. The calibration plot was calculated by 500 repetitions of bootstrap resampling to assess the predictive accuracy of the PPCs. In addition, the AUC, sensitivity and specificity of the reported prediction models for PPCs (ASA class and the ARISCAT score), risk scores for morbidities after thoracic surgery (Eurolung1) and risk models obtained from the American Society of Thoracic Surgeons General Thoracic Surgery Database (of lung cancer and esophageal cancer) were evaluated and compared to those of above-established models.

All the data analyses were performed with SPSS V.25.0 software and STATA V.16.0 software.

## Results

### Baseline characteristics and incidence of PPC events

In the present study, 475 patients admitted to the ICU after noncardiac thoracic surgery were recruited. The median (interquartile range, IQR) age was 58(49–68), and 72% (342/475) were male. At least one PPC occurred in 171 patients (36.0%), with 276 PPC events. Patients with PPCs had more cases of hypertension, a higher ASA class and lower albumin level (*p* < 0.05; Table 1). There were more cases of emergency surgery, more cases of surgery in the mediastinum or thoracic wall, and more cases of open chest surgery in patients with PPCs (*p* < 0.05; Table 1). Regarding the data during the ICU stay, most of data differed between patients with and without PPCs (Table 2 and Supplemental Table 1). After extubation, the patients who developed PPCs were in a worse condition and needed higher FiO2, and had lower PaO2 and pH according to arterial blood gas (ABG) analysis (Table 2). There were no significant differences in baseline characteristics between the development cohort and the validation cohort except for the median age (Supplemental Table 2).

**Table 1:** Baseline characteristics

Variables		At least 1 PPC *n* = 171(36.00%)	No PPC *n* = 304(64.00%)	*P* value
Age, median (IQR)		61(50–68)	57(48–68)	0.16
sex, n (%)	male	127(75.15)	215(70.26)	0.26
BMI > 25, n (%)		46(27.22)	78(25.49)	0.53
Comorbidities				
COPD, n(%)		52(30.77)	84(27.45)	0.44
Pneumonia, n(%)		20(11.83)	32(10.46)	0.65
Hypertension, n(%)		40(23.67)	45(14.71)	0.015*
Smoking, n(%)		71(42.01)	132(43.14)	0.79
ASA class, n(%)	>=3	84(66.67)	105(43.03)	
	< 3	42(33.33)	139(56.97)	0.027*
ARISCAT score, median(IQR)		50(50–58)	50(43–50)	< 0.001***
Eurolung1(2016), median(IQR)		5 (3–6)	5 (3–6)	0.13
Eurolung1(2019), median(IQR)		3(2.5-5)	2.5(2.5-5)	0.058
AST(U/L), median(IQR)		21 (16–29)	18 (15–23)	< 0.001***
Albumin(g/L), median(IQR)		40.85(35.6–44.1)	42(39-44.7)	0.006**
Urine(mmol/L), median(IQR)		5.5(4.5-7)	5.1(4.1–6.2)	0.005**
Pulmonary function, n(%)	normal	21(16.03)	50(25.91)	
	abnormal	39(29.77)	63(32.64)	0.053
FEV1(L), median(IQR)		2.5(1.85–3.27)	2.61(2.15–3.28)	0.46
FEV1/FVC, median(IQR)		0.79(0.71-0.84)	0.78(0.71-0.84)	0.81
FEV1-ppo(L), median(IQR)		1.88(1.33–2.66)	1.87(1.39–2.27)	> 0.99
Echocardiography, n(%)	normal	32(19.05)	65(21.74)	
	abnormal	43(25.60)	76(25.42)	0.84
Abnormal electrocardiogram(ECG), n(%)		40(38.83)	73(34.11)	0.019*
Intra-operative variables				
Surgery type, n(%)	emergency	46(26.90)	32(10.60)	
	elective	125(73.10)	270(89.40)	< 0.001***
Duration of surgery, n(%)	<=2 h	18(10.65)	57(19.32)	
	2–3 h	22(13.02)	43(14.58)	
	> 3 h	129(76.33)	195(66.10)	0.033*
Surgical site, n(%)	lung	26(15.20)	67(22.19)	
	mediastinum	57(33.33)	90(29.80)	
	esophagus	80(46.78)	141(46.69)	
	thoracic wall	8(4.68)	4(1.32)	0.046*
Surgical approach, n(%)	VATS/RATS	94(54.97)	218(72.19)	
	open chest	71(41.52)	76(25.17)	
	others	6(3.51)	8(2.65)	0.001**
Blood transfusion, n(%)		19(19.79)	14(9.66)	0.025*
Prophylactic antibiotics, n(%)		77(80.21)	131(90.34)	0.056
Liquid(mL), median(IQR)		2500(1550–3200)	2000(1100–2700)	0.015*

Data were presented as count and percentage or median and interquartile range(IQR);

Abbreviations: ARISCAT: The Assess Respiratory Risk in Surgical Patients in Catalonia; ASA: American Society of Anaesthesiologists physical status classification system; AST: aspartate transaminase; BMI: body mass index; CHD: coronary heart diseases; COPD: chronic obstructive pulmonary diseases; FEV1: Forced Expiratory Volume in one second; FEV1-ppo: postoperative FEV1; FVC: forced vital capacity; PPC: postoperative pulmonary complication; RATS: robot-assisted thoracic surgery; VATS: video-assisted thoracic surgery

***:*p* &lt; 0.001,**:*p* &lt; 0.01, *:*p* &lt; 0.05

**Table 2:** Postoperative variables during intensive care unit stay

Variables median(IQR)	At least 1 PPC *n* = 171(36.00%)	No PPC *n* = 304(64.00%)	*P* value
APACHE II score	12 (8–17)	11 (7–15)	0.015*
Heart rate	83.5(74.5–97.5)	79(70–90)	< 0.001***
FiO2	0.40(0.40-0.50)	0.40(0.40-0.40)	< 0.001***
PaO2(mmHg)	106.25(82.8-135.7)	124(95.3-157.8)	< 0.001***
PaCO2(mmHg)	43.1(39-47.8)	41.8(37.2–45.5)	0.012*
pH	7.34(7.31–7.38)	7.36(7.33–7.39)	< 0.001***
Lactate(mmol/L)	1.9(1.4–2.9)	1.7(1.3–2.4)	0.015*
Hemoglobin(g/L)	114(98–127)	121(109–132)	< 0.001***
Platelet(10^9^/L)	147(105–211)	164(128-209.5)	0.043*
AST(U/L)	34(24–53)	28(20–42)	< 0.001***
Albumin(g/L)	30.6(25.5–33.4)	33.2(29.5–36.9)	< 0.001***
C-reactive protein(mg/L)	7.84(4.41–85.9)	4.16(2.135–8.645)	< 0.001***
Procalcitonin(ng/mL)	0.175(0.07-0.73)	0.06(0.03–014)	< 0.001***
Interleukin-6(pg/mL)	334(189–636)	247.75(103-454.5)	0.003**
Prothrombin time(s)	12(11-13.5)	11.6(10.9–12.4)	0.002**
APTT(s)	28.35(25.4–33.1)	27.3(25.1–29.9)	0.005**
International normalized ratio	1.075(0.985 − 1.2)	1.04(0.98-1.11)	0.007**
Thrombin time(s)	17.4(16.1–18.4)	17.7(16.9–19.1)	0.002**
D-dimmer(ug/mL)	3.07(1.88–4.99)	1.89(0.955 − 3.76)	< 0.001***
P/F, PaO2/FiO2	245.5(178.75-307.75)	300.5(232.25–377)	< 0.001***
PEEP(cmH2O)	5(5–6)	5(5–6)	0.019*
Ventilation strategy after extubation, n(%)			
Conventional oxygen, n(%)	103(60.23)	265(87.17)	
HFNO, n(%)	24(14.04)	19(6.25)	
Non-invasive, n(%)	38(23.17)	13(4.35)	
others, n(%)	1(0.61)	1(0.33)	< 0.001***
HFNO, n(%)	24(14.04)	19(6.25)	0.005**
FiO2	0.385(0.33-0.41)	0.33(0.33-0.40)	< 0.001***
SpO2	1(0.99 − 1)	1(1–1)	0.011*
PaO2(mmHg)	95.15(77.3-115.4)	106.1(85.5-142.3)	< 0.001***
PaCO2(mmHg)	40.6(37.55–44.5)	41.5(38.3–45.2)	0.25
pH	7.39(7.36–7.44)	7.38(7.36–7.41)	0.023*
Liquid balance, mL Median(IQR)			
pod1	673(112–1245)	537.5(116–1124)	0.22
pod2	534(-218-1204.5)	434(-130-1210)	0.93
pod3	123.55(-480-986.5)	190(-155-854)	0.43

Data were presented as mean (standard deviation, SD) or median(interquartile range, IQR) or count and percentage;

Abbreviations: APACHE II: Acute Physiology and Chronic Health Evaluation II; APTT: activated partial prothrombin time; AST: aspartate transaminase; FiO2: fraction of inspiration oxygen; HFNO: high flow nasal oxygen; ICU: intensive care unit; MV: mechanical ventilation; PaCO2: arterial carbon dioxide tension; PaO2: pulmonary arterial oxygen tension; PEEP: positive end-expiratory pressure; P/F: oxygen ratio: PaO2/FiO2; pod: postoperative days; PPC: postoperative pulmonary complication; WBC: white blood cell counts

***:*p* < 0.001,**:*p* < 0.01, *:*p* < 0.05

In patients who developed PPCs, 77 patients (15.58%) developed multiple PPCs: 53 patients (11.16%) had two PPCs, and 21(4.42%) had three PPCs (Fig. 1, B), with no patients developing four or more PPCs. The most common PPC was pneumonia (153/475, 32.21%), followed by respiratory failure (58/475, 12.21%), pleural effusion (47/475, 9.89%), ARDS (6/476, 1.26%), pulmonary embolism (5/475, 1.05%), pneumothorax (4/475, 0.84%), and bronchospasm (3/475, 0.63%) (Fig. 1, C). Most of the PPCs occurred during the first three pods (138/171, 80.70%), especially the first two pods (104/171, 60.82%) (Fig. 1, D).

### Patient outcomes

As shown in Table 3, patients who developed at least one PPC had a longer duration of mechanical ventilation (h, median [IQR], 13.8[8.18–38.33] vs. 8.9[3.36-14.00], *p* < 0.001), length of ICU stay (d, median [IQR], 3[2–6] vs. 2[2–2], *p* < 0.001) and LOS (d, median [IQR], 14[10–22] vs. 11[7–14], *p* < 0.001). In addition, patients with at least one PPC were more likely to undergo reintubation (20/171[11.70%] vs. 4/304[1.32%], *p* = 0.047). Differences in specific PPCs, the cumulative number of PPCs and time of the occurrence of PPCs were also compared (Supplemental Tables 3 and Table 4). Moreover, there were no significant differences in the primary or secondary outcomes between the development cohort and the validation cohort (Supplemental Table 2).

**Table 3:** PPC and secondary outcomes

Secondary outcomes	At least 1 PPC *n* = 171(36.00%)	No PPC *n* = 304(64.00%)	Total *N* = 475	*P* value
Duration of MV, h Median(IQR)	13.80(8.18–38.33)	8.99(3.36–14.01)	10.36(4.32–17.01)	< 0.001^***^
ICU stay, d Median(IQR)	3 (2–6)	2(2–2)	2(2–3)	< 0.001^***^
LOS, d Median(IQR)	14 (10–22)	11 (7–14)	11 (8–16)	< 0.001^***^
Re-intubation, n(%)	20(11.70)	4(1.32)	24(5.05)	0.047^*^
Transfusion of blood or blood component, n(%)	10(5.85)	9(2.96)	19(4.00)	0.12
Transfusion of albumin n(%)	49(28.65)	45(14.80)	94(19.79)	< 0.001^***^
CRRT, n(%)	4(2.34)	0(0)	4(2.34)	0.016^*^
Rescue during ICU stay, n(%)	17(9.94)	6(1.97)	23(4.84)	< 0.001^***^
Re-surgery, n(%)	10(5.85)	11(3.62)	21(4.42)	0.26
ICU readmission, n(%)	9(5.26)	13(4.28)	21(4.42)	0.50
In-hospital mortality n(%)	3(1.75)	3(0.99)	6(1.26)	0.47
Automatic discharge, n(%)	11(6.43)	7(2.30)	18(3.79)	0.024^*^

Data were presented as count and percentage or median (interquartile range, IQR);

Abbreviations: CRRT: continuous renal replacement therapy; ICU: intensive care unit; LOS: length of hospital stay; MV: mechanical ventilation; PPC: postoperative pulmonary complication

***:*p* < 0.001,**:*p* < 0.01, *:*p* < 0.05

**Table 4:** Multivariate logistic regression results (backward elimination, *p* < 0.05)

Variables		Odds ratio	*P* value	95% CI
ASA class > = 3		2.03	0.011^*^	1.17–3.50
Albumin		0.93	0.025^*^	0.88-0.99
Surgery type		0.56	0.017^*^	0.35-0.90
Surgery site		1.50	0.026^*^	1.05–2.13
PaO2		0.98	0.000^***^	0.97-0.99
Albumin at ICU admission		0.89	0.003^**^	0.82-0.96
Duration of MV		1.04	0.000^***^	1.02–1.06

Abbreviations: ASA: American Society of Anesthesiologists; CI: confidence interval; ICU: intensive care unit; MV: mechanical ventilation; PaO2: pulmonary arterial oxygen tension

***:*p* < 0.001,**:*p* < 0.01,*:*p* < 0.05

### Development and validation of nomograms

**Fig. 2:**
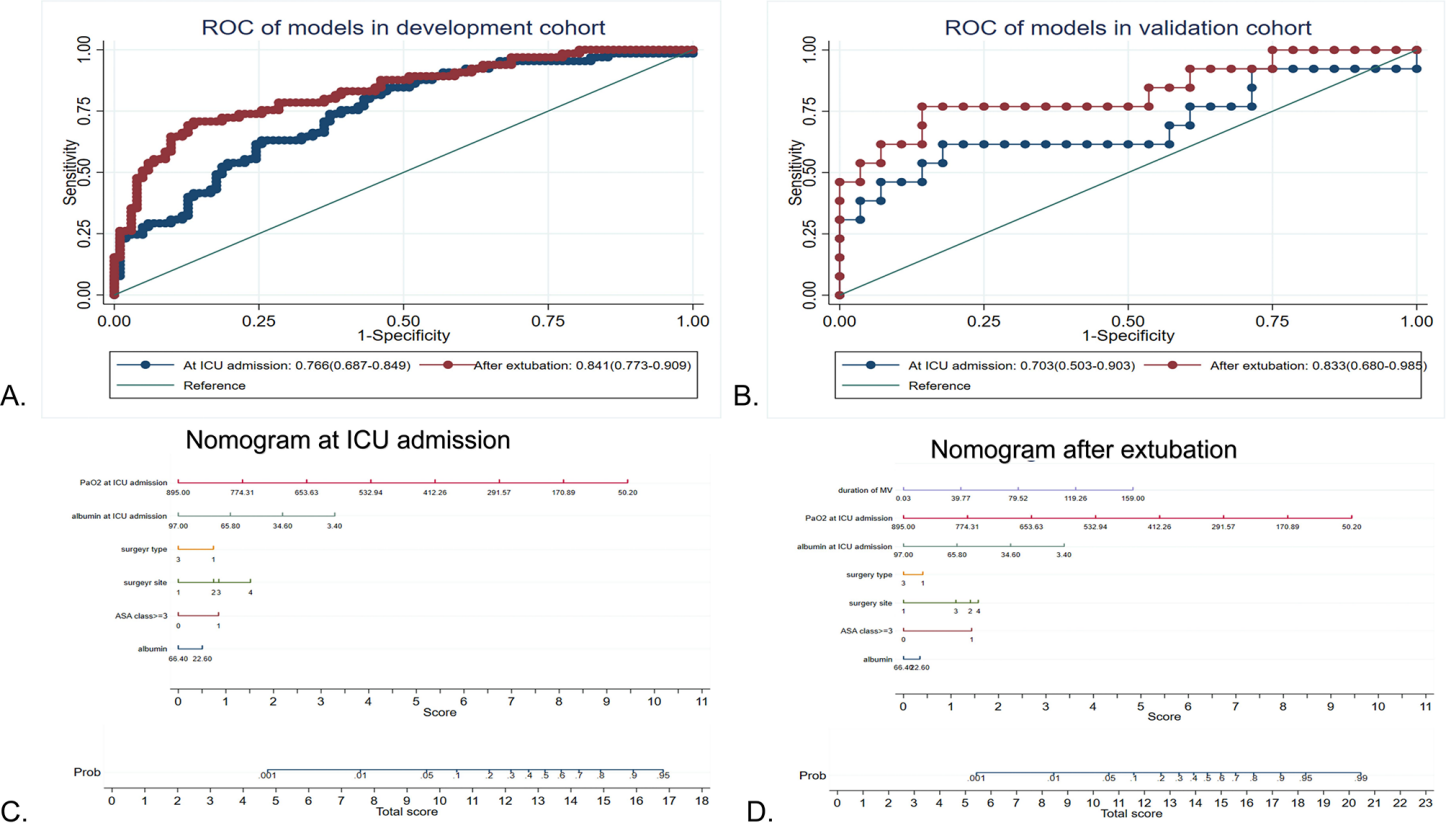
(**A**) ROC curve for PPCs, at ICU admission and after extubation in the development cohort; (**B**) ROC curve for PPCs, at ICU admission and after extubation in the validation cohort; (**C**) Nomogram for PPCs, at ICU admission; (**D**) Nomogram for PPCs, after extubation. To estimate the risk of PPCs, mark patient values at each axis, draw a straight line perpendicular to the point axis, and sum the points for all variables. Next, mark the sum on the total point axis and draw a straight line perpendicular to the risk axis. PaO2, mmHg; albumin, g/L; duration of MV, h Abbreviations: ASA: American Society of Anesthesiologists; ICU: intensive care unit; MV: mechanical ventilation; PaO2: pulmonary arterial oxygen tension; PPC: postoperative pulmonary complication

Univariate logistic regression analysis was applied to evaluate the association of each variable with the PPC, shown in Supplemental Table 5. A *p*-value less than 0.1 was considered a candidate predictor. Multivariate logistic regression (stepwise backward elimination, *p* < 0.05) was subsequently used to exclude confounding factors. Hence, an ASA class higher than 3, preoperative albumin level, surgery site, surgery type and albumin level at ICU admission, PaO2 at ICU admission, and duration of mechanical ventilation were found to be significantly and independently associated with PPC (Table 4). Two models were subsequently constructed; one at ICU admission, and one after extubation. The ROC curves and nomograms are shown in Fig. 2.

**Fig. 3:**
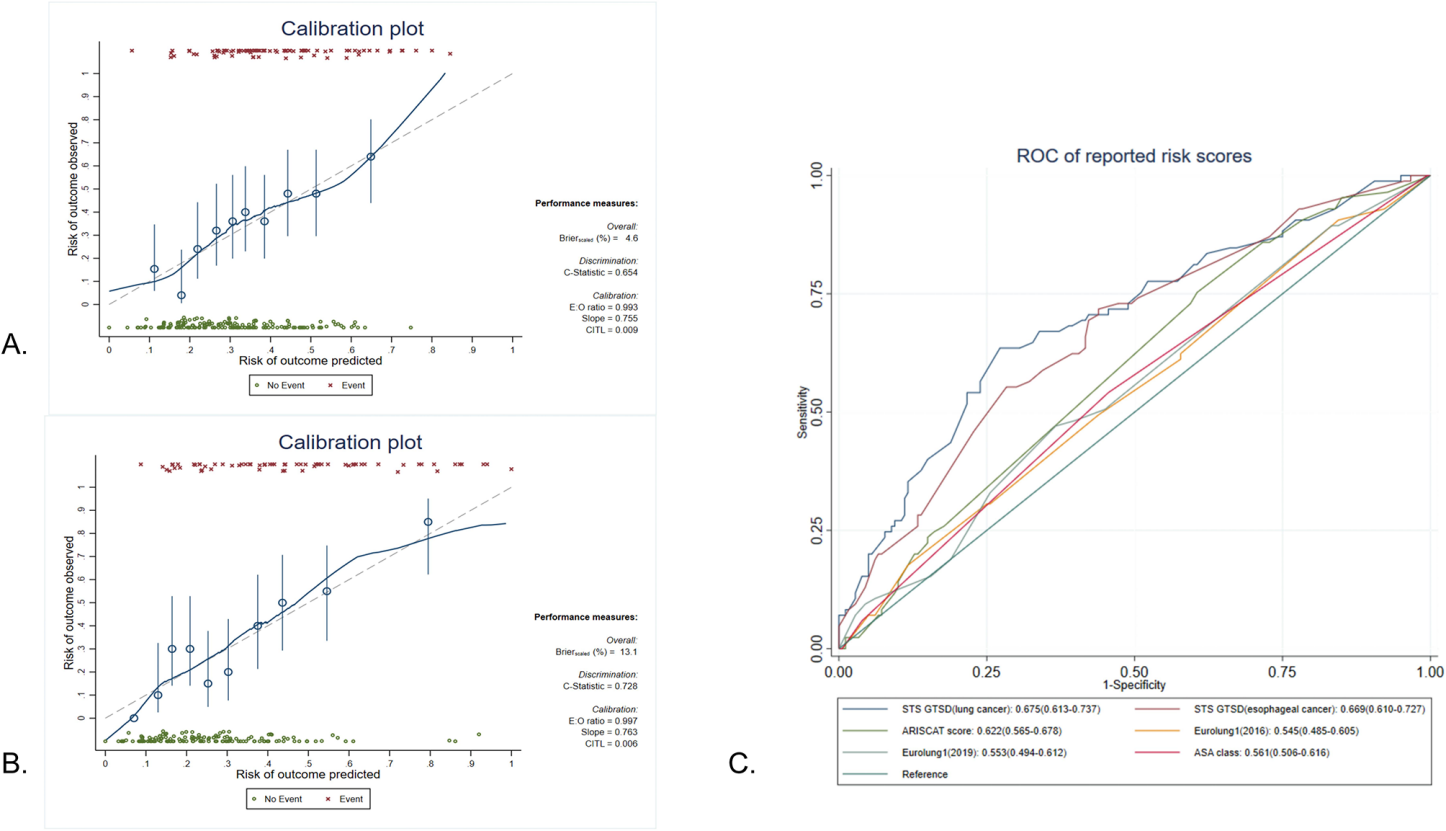
(**A**) Calibration plot of the nomogram for predicting PPC, at ICU admission; (**B**) Calibration plot of the nomogram for predicting PPC, after extubation. The X-axis is the predicted probability of PPC; the Y-axis is the observed probability of PPC. (**C**) ROC curve of reported risk models for PPCs. The reported risk models included the ASA class, the ARISCAT score, the Eurolung1 score (2016), and the Eurolung1 score(2019), obtained from the STS GTSD (of lung cancer and esophageal cancer) Abbreviations: ARISCAT: The Assess Respiratory Risk in Surgical Patients in Catalonia; ASA: American Society of Anaesthesiologists; Eurolung1: European risk models for morbidity to predict outcome following anatomic lung resections; ICU: intensive care unit; PPC: postoperative pulmonary complication; STS GTSD: The Society for Thoracic Surgeons General Thoracic Surgery Database

In the development cohort, at ICU admission, an ASA class higher than 3, preoperative albumin level, surgery site and surgery type, PaO2, and albumin levels formed the first model, whose AUC was 0.766 (95% CI, 0.687 to 0.849), with a sensitivity of 0.71 and specificity of 0.60(Goodness of fit test showed x^2^ = 247.81, *p* = 0.37). After extubation, the duration of MV was added to the second model, whose AUC was 0.841(95% CI,0.773 to 0.909), with a sensitivity of 0.75 and specificity of 0.83(Goodness of fit test showed x^2^ = 131.55, *p* = 0.26) (Fig. 2A). To assess the predictive accuracy, the calibration plot was calculated by 500 repetitions of bootstrap resampling. Both models showed excellent predictive value. At ICU admission, the Brier score (%) was 4.6, and the C-statistic was 0.654; after extubation, the Brier score (%) was 13.1 and the C-statistic was 0.728(Fig. 3A, B). The two models were further tested in the validation cohort. The AUC at ICU admission was 0.703(95% CI, 0.503 to 0.903) and after extubation was 0.833(95% CI, 0.680 to 0.985) (Fig. 2B). Both models performed well in the development cohort and the validation cohort, thus they could be used at different times to evaluate the risk of PPC and enhance the postoperative care of patients.

Since reported risk scores for PPCs are not widely used in clinical practice, we aimed to develop simple and effective models for PPCs. Then the ROC curves of our models with those of the ASA class, the ARISCAT score, the Eurolung1 score(2016), Eurolung1 score(2019), and risk models from the STS GTSD (of lung cancer and esophageal cancer) were drawn. The ROC curves and AUC of the reported risk models were shown in Fig. 3C. The sensitivity and specificity of our established models and reported risk scores are shown in Supplemental Table 6. These findings indicated that our established models were more effective than the reported risk models and had excellent sensitivity and specificity.

## Discussion

As expected, ICU patients after noncardiac thoracic surgery were prone to develop PPCs, with a percentage of 36.0%, which is higher than that reported previously [1, 4, 15]. Our findings are consistent with a multicenter prospective observational study, in which the percentage of PPC was 33.4% [4]. However, it enrolled high risk patients. Current studies have shown that ICU admission is a poor outcome of PPC [4], so ICU patients are at high risk of PPCs. According to Tables 1 and 2, ASA class higher than 3 and the APACHE II score significantly differed between the PPC group and no PPC group. These findings demonstrated that patients with PPCs had worse organ function at admission, leading to more frequent PPC events and worse outcomes.

In line with prior studies, the current study confirmed that patients developed at least one PPC had a longer duration of mechanical ventilation, longer length of ICU stay, longer LOS, and a greater rate of reintubation [2, 4, 5, 13, 17]. There was no difference in in-hospital mortality between the two groups, due to the few events documented and the significant difference in automatic discharge. In addition, unlike in prior studies, the rise in the number of PPCs was associated with worse outcomes [1, 5]. This study showed that the number of PPCs had no impact on the duration of mechanical ventilation, or the length of ICU stay or LOS. In this study, most of the PPCs occurred during the first three pods, but the time of occurrence of PPCs did not influence clinical outcomes (Supplemental Table 3).

The current study showed that the most common PPC occurring in ICU patients after noncardiac thoracic surgery was pneumonia, which included respiratory infection and inspiration pneumonia. After major surgery, hypoxemia is common [32]. Therefore, most patients admitted to the ICU after surgery are always intubated and under mechanical ventilation. Ventilation-induced lung injury has been increasingly common in the ICU over the years, and leads to poor outcomes [33]. Thus, postoperative ICU patients are more likely to develop pneumonia.

There were plenty of studies highlighting the effects of intraoperative factors on PPC and clinical outcomes. In this study, patients who underwent emergency surgery, surgery in the mediastinum, esophagus, or thoracic wall, open chest surgery, or surgery lasting more than three hours had a greater incidence of PPCs, which is consistent with original articles [2, 15, 17]. VATS or RATS has been confirmed to have better short-term and long-term outcomes than thoracotomy [22, 23, 34]. Similar results were found in this study.

Moreover, several different risk factors for PPCs were identified in this study. During the preoperative phase, an ASA class higher than 3 and a lower preoperative albumin level were independently associated with PPCs, consistent with previous studies [3, 15, 24, 35]. Interestingly, there were no differences in age, BMI, SpO2, history of pulmonary infection or COPD, smoking status, the Brinkman index, or abnormal lung function [3, 36]. However, the intraoperative factors were the same as those reported, for the surgery site and surgery type. Patients who underwent emergency surgery or surgery in the mediastinum or thoracic wall were more likely to develop PPCs [15, 28]. It is innovative in this study, we found that PaO2, albumin level, and the duration of MV during the ICU stay were independently associated with the occurrence of PPCs, which was different from what was observed in original PPC articles recruiting postoperative ICU patients [4, 11]. At ICU admission, patients with lower PaO2 and albumin levels were prone to develop PPCs [17, 37]. The risk of PPCs augmented with the increase in the duration of mechanical ventilation [38]. Our findings illustrated the effect of PaO2 and duration of mechanical ventilation on the occurrence of PPCs, which was rarely reported previously. Therefore, we developed prediction nomograms for PPCs. To comprehensively evaluate the risk of PPCs in ICU patients, two prediction models were constructed based on the timeline after patients were admitted to the ICU. At ICU admission, the AUC was 0.766 (95% CI, 0.687 to 0.845). This could help identify high-risk patients in developing PPCs at ICU admission and improve postoperative management. After extubation, the AUC was 0.841 (95% CI,0.773 to 0.909). It is possible that those patients at high risk of PPCs at ICU admission were at a lower risk level after extubation. As the duration of mechanical ventilation was independently associated with PPCs, the model including the duration of MV was more precise. Concerning those patients reevaluated at high risk of PPCs after extubation, appropriate ventilation strategies could be administrated in time. Both models were well calibrated, showing good Brier score and C-statistic values. These models performed well in the validation cohort. Among these, the predictive value of our models with reported risk scores for PPC and morbidities after thoracic surgery were compared. With similar postoperative morbidities [1, 26–28, 39], these models might be effective in this study. Interestingly, the highest AUC of the reported risk models was obtained from the STS GTSD (for lung cancer), which was 0.675(0.613 to 0.737), followed by that obtained from the STS GTSD (for esophageal cancer), the ARISCAT score, the ASA class, the Eurolung1(2019) and the Eurolung1(2016). This indicated that our established models had the highest AUC, and could be considered the most effective prediction models (Supplemental Table 6). Considering the sensitivity, our established models performed better than most of the reported models, other than the ARISCAR score with the highest sensitivity (0.78). Regarding the specificity, the risk model after extubation had the highest specificity, following the risk model obtained from the STS GTSD (for lung cancer)(0.70). In conclusion, it indicated that our prediction models are more effective than previously reported risk models and have higher sensitivity and specificity. This might due to different enrolled populations and different risk factors identified. In this study, there were few lung sections and few available FEV1-ppo values (Table 1), leading to incomplete aggregated scores of Eurolung1. Several predictors were identified based on the American Society of Thoracic Surgeons General Thoracic Surgery Database. In addition to age, sex and type of surgery, the other predictors also included FEV1-ppo, steroid use and specific surgical procedures, which are different from our results. In addition, the reported risk models include only preoperative and intraoperative variables [1, 26, 27, 39, 40], and we also included ICU parameters during the postoperative phase.

This study has several strengths. First, to the best of our knowledge, this is the first study of PPC to enroll ICU patients after non-cardiac thoracic surgery. Previous PPC studies considered ICU admission as a complication, and those studies enrolled ICU patients focused on hepatectomy, on-pump cardiac surgery or noncardiothoracic surgery, rarely on non-cardiac thoracic surgery. Second, few PPC studies have enrolled patients who underwent noncardiac thoracic surgery. The tumour screening and cases of surgery at an early stage have been increasing these years, and the cases of VATS/RATS are increasingly common. Novel risk factors for PPCs emerged. Third, this study identified several novel risk factors for PPCs in ICU patients and established new prediction models for PPCs. A lower PaO2 level at ICU admission and a longer duration of mechanical ventilation were newly found to be independently associated with PPCs. Moreover, compared with reported risk models, the established models showed robust discrimination.

This study has several limitations. First, as a retrospective study, it was less effective than a prospective study. Second, the risk factors and prediction models in this study were applicable only to postoperative ICU patients after noncardiac thoracic surgery, and not to all postoperative patients. Due to the exclusion of patients whose length of ICU stay less than 24 h, the representativeness and validity of this study is limited. This could lead to selection bias, and severe cases of PPCs could be potentially underestimated. In addition, the intraoperative data were not completely documented, leading to defects in risk factors. Third, although the prediction models established in this study had high sensitivity and specificity, due to the modest sample size, the accuracy of the risk models needs to be further tested in larger population-based prospective studies and different enrolled populations. Despite these limitations, since there is no prediction model for PPC widely used, we hope that our models could help clinical physicians identify high-risk patients in developing PPCs and ameliorate the outcomes of postoperative ICU patients.

## Conclusion

In the present study, we found that ICU patients after noncardiac thoracic surgery were at high risk of developing PPCs. PPC significantly increased the duration of mechanical ventilation, length of ICU stay, LOS, and rate of reintubation. The ASA class, preoperative albumin level, surgery site, surgery type, PaO2 and albumin level at ICU admission, and duration of MV were found to be independent risk factors of PPCs. Then we constructed effective prediction nomograms with excellent sensitivity and specificity. These risk models were more accurate than reported risk models and could help assess the individual risk of PPC and improve the postoperative management of critical patients after noncardiac thoracic surgery.

### Electronic supplementary material

Below is the link to the electronic supplementary material.

Supplementary Material 1

Supplementary Material 2

## Data Availability

The datasets used and analysed during the current study are available from the corresponding author upon reasonable request.

## Abbreviations

ARDS: Acute respiratory distress syndrome
ARISCAT: The Assess Respiratory Risk in Surgical Patients in Catalonia
ASA: American Society of Anesthesiologists
AUC: Area under curve
BMI: Body mass index
CRRT: Continuous renal replacement therapy
Eurolung1: European risk models for morbidity to predict outcome following anatomic lung resections
FEV1-ppo: Postoperative forced expiratory volume in one second
ICU: Intensive care unit
LOS: Length of hospital stay
MV: Mechanical ventilation
PaCO2: Arterial carbon dioxide tension
PaO2: Pulmonary arterial oxygen tension
PEEP: Post end-expiratory pressure
POD: Postoperative days
PPC: Postoperative pulmonary complication
RATS: Robot-assisted thoracic surgery
ROC: Receiver Operating Characteristic curve
STS GTSD: The Society for Thoracic Surgeons General Thoracic Surgery Database
VATS: Video-assisted thoracic surgery
