# Supplementary material for: Prediction models for postoperative pulmonary complications in intensive care unit patients after noncardiac thoracic surgery
Source: BMC Pulm Med. 2024 Aug 29;24:420. doi: 10.1186/s12890-024-03153-z (PMC11360767; doi:10.1186/s12890-024-03153-z)
Supplement: Supplementary file 1 — Supplementary Material 1 [file 12890_2024_3153_MOESM1_ESM.docx]

PPC（Postoperative pulmonary complications）：

| Outcomes | definitions |
| --- | --- |
| Pneumonia (Respiratory infection,Aspiration pneumonitis) | Antibiotics for suspected infection with one or more of the following: new or changed sputum, new or changed lung opacities, fever, white blood cell count >12*10^9^/ L  Acute lung injury after inhalation of regurgitated gastric contents |
| Respiratory failure | Postoperative PaO2 <8 kPa (60 mm Hg) on room air, a PaO2 :FIO2 ratio <40 kPa (300 mm Hg), or arterial oxyhaemoglobin saturation measured with pulse oximetry <90% and requiring oxygen therapy |
| Pleural effusion | Pleural effusion requiring thoracocentesis |
| Pneumothorax | Pneumothorax requiring thoracocentesis |
| Bronchospasm | Clinical diagnosis resulting in change in therapy89 Refractory wheeze requiring parenteral drugs in addition to preoperative regimen |
| ARDS | Ventilated, bilateral infiltrates on CXR, PaO2 :FIO2 <300 |
| Pulmonary embolism | CTA diagnosis |

Baseline characteristics

| name | | | 🞎🞎🞎🞎 | | |
| --- | --- | --- | --- | --- | --- |
| Age | | | 🞎🞎 | | |
| Sex | | |  | | |
| ID number | | |  | | |
| height | | | cm | | |
| weight | | | 🞎🞎🞎kg | | |
| BMI,kg/m2 | | | 🞎Underweight (< 18.5)  🞎Normal weight (18.5-24.9)  🞎Overweight (25-29.9) | | |
| Admission time | | | 🞎🞎🞎🞎年🞎🞎月🞎🞎日 | | |
| Discharge time | | | 🞎🞎🞎🞎年🞎🞎月🞎🞎日 | | |
| Past history | | |  | | |
| Respiratory system | | | 🞎COPD 🞎asthma  🞎pneumonia（<30d）🞎OSAHS 🞎TB 🞎lung tumor | | |
| Circulatory system | | | 🞎hypertension 🞎CHD 🞎heart failure  🞎congenital heart disease 🞎valve disease 🞎pulmonary hypertension | | |
| Digestive system | | | 🞎hepatitis 🞎cirrhosis | | |
| Urological system | | | 🞎chronic kidney disease | | |
| Endocrine or metabolism | | | 🞎diabetes 🞎cushing syndrome 🞎hyperlipidemia 🞎thyroid disease | | |
| Others | | | 🞎anemia 🞎epilepsy 🞎cerebral infarction | | |
| Surgery history | | |  | | |
| Personal history | | |  | | |
| Smoking history | | | 🞎no 🞎yes | | |
| smoking index | | | number/d*y | | |
| Smoking cessation | | | 🞎yes 🞎no | | |
| Smoking cessation time | | |  | | |
| Drinking history | | | 🞎no 🞎yes | | |
|  | | |  | | |
| symptoms | |  | | | |
| cough | | 🞎yes 🞎no | | | |
| sputum | | 🞎yes 🞎no | | | |
| characteristics | | 🞎sticky 🞎sparse  colors | | | |
| dyspnea | | 🞎yes 🞎no | | | |
| Fever(>=37.3℃) | | 🞎yes 🞎no | | | |
| Chest pain | | 🞎yes 🞎no | | | |
| hemoptysis | | 🞎yes 🞎no | | | |
| Night sweat | | 🞎yes 🞎no | | | |
| ASA class | | 🞎1 🞎2 🞎3 🞎4 🞎5 | | | |
| ARISCAT score | | 🞎low risk(<=26) 🞎moderate risk(26-45) 🞎high risk(>45) | | | |
| Diagnosis at admission | |  | | | |
|  | hemoglobin | | |  |  |
|  | WBC | | |  |  |
|  | neutrophils | | |  |  |
|  | platelet | | |  |  |
|  | urine | | |  |  |
|  | creatinine | | |  |  |
|  | eGFR | | |  |  |
|  | CRP | | |  |  |
|  | ESR | | |  |  |
|  | PCT | | |  |  |
|  | IL-6 | | |  |  |
|  | ALT | | |  |  |
|  | AST | | |  |  |
|  | albumin | | |  |  |
|  | Unconjugated bilirubin | | |  |  |
|  | Conjugated bilirubin | | |  |  |
| ECG | 🞎none 🞎normal 🞎abnormal | | |  |  |
| echocardiography | 🞎none 🞎normal 🞎abnormal | | |  |  |
|  | Left ventricular EF | | |  |  |
|  | Right ventricular EF | | |  |  |
| Lung function test | 🞎none 🞎normal 🞎abnormal | | |  |  |
|  | FEV1,L | | |  |  |
|  | FVC,L | | |  |  |
|  | FEV1/FVC | | | 🞎normal 🞎mild(>=70%)  🞎moderate(60-69%) 🞎severe(<50%) |  |
|  | PEF,L/s | | |  |  |
|  | MEF75,L/s | | |  |  |
|  | MEF50,L/s | | |  |  |
|  | MEF25,L/s | | |  |  |
|  | DLCO SB,ml/min/mmHg | | |  |  |
| 6min walking test (6MWT) | 🞎none 🞎normal 🞎abnormal | | |  |  |
| X-ray | 🞎none 🞎normal 🞎abnormal | | |  |  |
| CT | 🞎none 🞎normal 🞎abnormal | | |  |  |
| ABG | 🞎none 🞎normal 🞎abnormal | | |  |  |
|  | PaO2,mmHg | | |  |  |
|  | PaCO2,mmHg | | |  |  |
|  | pH | | |  |  |
|  | SaO2,% | | |  |  |
|  | HCO3- | | |  |  |
|  | Lac | | |  |  |
|  | glucose | | |  |  |
| Etiology test | Sputum Culture | | | 🞎none 🞎normal 🞎abnormal |  |
|  | Virus | | | 🞎none 🞎normal 🞎abnormal |  |
|  | GM test | | | 🞎none 🞎normal 🞎abnormal |  |
|  | BALF | | | 🞎none 🞎normal 🞎abnormal |  |

Intraoperative phase:

| Surgery approach | 🞎VATS/RATS 🞎open chest 🞎others |
| --- | --- |
| Surgery site | 🞎lung 🞎mediastinum 🞎esophagus 🞎others |
| Surgery type | 🞎emergency 🞎limited 🞎elective |
| Duration of surgery | 🞎<=2h 🞎2-3h 🞎>3h |
| anesthesia | 🞎general 🞎local |
| Epidural anesthesia | 🞎yes 🞎no |
| Duration of anesthesia |  |
| Ventilation mode | 🞎one-lung  🞎dual-lung |
| Type of intubation tube | 🞎6 🞎6.5 🞎7 🞎7.5 🞎8 |
| Prophylatic antibiotics | 🞎yes 🞎no |
| Blood Transfusion | 🞎yes 🞎no |
| liquid |  |

Postoperative phase

| ICU admission time |  |
| --- | --- |
| ICU exit time |  |
| Diagnosis at ICU admission |  |
| APACHEII score |  |
| SOFA score |  |

| ICU | | D0 | D1 | D2 | D3 | D4 | D5 | D6 | D7 | D14 | D28 |
| --- | --- | --- | --- | --- | --- | --- | --- | --- | --- | --- | --- |
| Ventilation mode | 🞎1.A-C（VC） 🞎2.A-C（PC）  🞎3.SIMV（PC）+PS 🞎4.SIMV（VC）+PS 🞎5.spontaneous  🞎6.other |  |  |  |  |  |  |  |  |  |  |
| Tidal volume,mL |  |  |  |  |  |  |  |  |  |  |  |
| PI |  |  |  |  |  |  |  |  |  |  |  |
| PS |  |  |  |  |  |  |  |  |  |  |  |
| PEEP,cmH2O |  |  |  |  |  |  |  |  |  |  |  |
| Setting RR,bpm |  |  |  |  |  |  |  |  |  |  |  |
| FiO2,% |  |  |  |  |  |  |  |  |  |  |  |
| SpO2,% |  |  |  |  |  |  |  |  |  |  |  |
| PetCO2,mmHg |  |  |  |  |  |  |  |  |  |  |  |
| MAP,mmHg |  |  |  |  |  |  |  |  |  |  |  |
| CVP,mmHg |  |  |  |  |  |  |  |  |  |  |  |
| PAWP,mmHg |  |  |  |  |  |  |  |  |  |  |  |
| HR,bpm |  |  |  |  |  |  |  |  |  |  |  |
| Monitoring RR |  |  |  |  |  |  |  |  |  |  |  |
| BP,mmHg |  |  |  |  |  |  |  |  |  |  |  |
| ABG at admission |  |  |  |  |  |  |  |  |  |  |  |
| PaO2,mmHg |  |  |  |  |  |  |  |  |  |  |  |
| paCO2,mmHg |  |  |  |  |  |  |  |  |  |  |  |
| pH |  |  |  |  |  |  |  |  |  |  |  |
| HCO3- |  |  |  |  |  |  |  |  |  |  |  |
| FiO2 |  |  |  |  |  |  |  |  |  |  |  |
| Lac |  |  |  |  |  |  |  |  |  |  |  |
| glucose |  |  |  |  |  |  |  |  |  |  |  |
| PaO2/FiO2,mmHg |  |  |  |  |  |  |  |  |  |  |  |
| Max Temperature |  |  |  |  |  |  |  |  |  |  |  |
| 24h liquid intake |  |  |  |  |  |  |  |  |  |  |  |
| 24h liquid output |  |  |  |  |  |  |  |  |  |  |  |
| Ultrafiltrate volume |  |  |  |  |  |  |  |  |  |  |  |
| Sputum volume |  |  |  |  |  |  |  |  |  |  |  |
| Phlegm characteristics | 🞎1.sticky phlegm  🞎2.sparse phlegm |  |  |  |  |  |  |  |  |  |  |
|  | 🞎1.red  🞎2.yellow 🞎3.pink 🞎4.white |  |  |  |  |  |  |  |  |  |  |
| bronchoscopy | 1.yes 2.no |  |  |  |  |  |  |  |  |  |  |
| Prone position | 1.yes 2.no |  |  |  |  |  |  |  |  |  |  |
| CRRT | 1.yes 2.no |  |  |  |  |  |  |  |  |  |  |
| Duration of CRRT |  |  |  |  |  |  |  |  |  |  |  |
| Lab tests | | | | | | | | | | | |
|  | hemoglobin |  |  |  |  |  |  |  |  |  |  |
|  | WBC |  |  |  |  |  |  |  |  |  |  |
|  | platelet |  |  |  |  |  |  |  |  |  |  |
|  | neutrophils |  |  |  |  |  |  |  |  |  |  |
|  | hct |  |  |  |  |  |  |  |  |  |  |
|  | ALT |  |  |  |  |  |  |  |  |  |  |
|  | AST |  |  |  |  |  |  |  |  |  |  |
|  | albumin |  |  |  |  |  |  |  |  |  |  |
|  | Unconjugated bilirubin |  |  |  |  |  |  |  |  |  |  |
|  | Conjugated bilirubin |  |  |  |  |  |  |  |  |  |  |
|  | urine |  |  |  |  |  |  |  |  |  |  |
|  | creatinine |  |  |  |  |  |  |  |  |  |  |
|  | eGFR |  |  |  |  |  |  |  |  |  |  |
|  | K+ |  |  |  |  |  |  |  |  |  |  |
|  | Na+ |  |  |  |  |  |  |  |  |  |  |
|  | Cl- |  |  |  |  |  |  |  |  |  |  |
|  | Ca2+ |  |  |  |  |  |  |  |  |  |  |
|  | CRP |  |  |  |  |  |  |  |  |  |  |
|  | ESR |  |  |  |  |  |  |  |  |  |  |
|  | PCT |  |  |  |  |  |  |  |  |  |  |
|  | IL-6 |  |  |  |  |  |  |  |  |  |  |
| 病原学结果  🞎1.未做 🞎2.正常 🞎3.异常 | 痰涂片/培养 |  |  |  |  |  |  |  |  |  |  |
|  | 呼吸道病毒十三项 |  |  |  |  |  |  |  |  |  |  |
|  | G/GM试验 |  |  |  |  |  |  |  |  |  |  |
|  | 肺泡灌洗液 |  |  |  |  |  |  |  |  |  |  |
| Ultrasound |  |  |  |  |  |  |  |  |  |  |  |
| echocardiography🞎1.none 🞎2.normal 🞎3.abnormal | Left ventricular EF |  |  |  |  |  |  |  |  |  |  |
|  | Right ventricular EF |  |  |  |  |  |  |  |  |  |  |
| Imaging progress |  |  |  |  |  |  |  |  |  |  |  |
| Procedures | rescue |  |  |  |  |  |  |  |  |  |  |
|  | resurgery |  |  |  |  |  |  |  |  |  |  |
|  | others |  |  |  |  |  |  |  |  |  |  |
| Major PPC | pneumonia |  |  |  |  |  |  |  |  |  |  |
|  | Respiratory failure |  |  |  |  |  |  |  |  |  |  |
|  | Pleural effusion |  |  |  |  |  |  |  |  |  |  |
|  | pneumothorax |  |  |  |  |  |  |  |  |  |  |
|  | bronchospasm |  |  |  |  |  |  |  |  |  |  |
|  | ARDS |  |  |  |  |  |  |  |  |  |  |
|  | Pulmonary emnolism |  |  |  |  |  |  |  |  |  |  |

| Time of extubation |  |
| --- | --- |
| Ventilation strategies after extubation | 🞎nasal tube 🞎mask 🞎HFNO 🞎noninvasive 🞎others |
| Flow rate of oxygen,L/min | 🞎>6L/min 🞎conventional |
| Conventional oxygen therapy(nasal tube and mask) | Flow rate： |
| HFNO | Flow rate： fraction of oxygen： |
| Noninvasive ventilation | 🞎S/T 🞎CPAP 🞎Other |
| IPAP,cmH2O |  |
| EPAP,cmH2O |  |
| FiO2 |  |
| Setting RR |  |
| Monitoring RR |  |
| ABG result |  |
| PaO2,mmHg |  |
| paCO2,mmHg |  |
| pH |  |
| HCO3- |  |
| FiO2 |  |
| Lac |  |
| glucose |  |
| HR |  |
| BP |  |
| Ventilation mode | 🞎volume controlled 🞎pressure controlled  🞎pressure support 🞎spontaneous  🞎other |
| Tidal volume,mL |  |
| Peak pressure,cmH2O |  |
| Plato pressure,cmH2O |  |
| PEEP,cmH2O |  |
| RR,bpm |  |
| FiO2,% |  |
| SpO2,% |  |
| MAP,mmHg |  |
| HR,bpm |  |

Outcomes

| Length of ICU stay,days |  |
| --- | --- |
| Unplanned ICU admission | 🞎yes 🞎no |
| Major PPC | 🞎无 🞎respiratory failure 🞎pneumonia 🞎pneumothorax 🞎pleural effusion 🞎ARDS 🞎bronchospasm |

| Re-intubation |  |
| --- | --- |
| duration of mechanical ventilation |  |
| Duration of noninvasive ventilation |  |
| ICU re-admission | 🞎yes 🞎no |
| ICU exit |  |
| ICU mortality | 🞎yes 🞎no |
| Reason of ICU exit | 🞎1.recovery and transfer to another department 🞎2.recovery and transfer to another hospital🞎3.recovery and hospital discharge 🞎4.deterioration and hospital discharge 🞎5.others___________ |
| discharge | 🞎yes 🞎no |
| Discharge time |  |
| In-hospital mortality | 🞎yes 🞎no |
| Reason of hospital discharge | 🞎1.automatic discharge 🞎2.recovery 🞎3.recovery and transfer to another hospital |
| Automatic discharge | 🞎1.deterioration 🞎2.financial problems 🞎3.transfer demand of relatives |

ARISCAT score

| Age |  |
| --- | --- |
| ≤50 years old | 0 points |
| 51 to 80 years old | 3 points |
| >80 years old | 16 points |
| Preoperative oxygen saturation |  |
| ≥96% | 0 points |
| 91 to 95% | 8 points |
| ≤90% | 24 points |
| Other clinical risk factors |  |
| Respiratory infection in the last month | 17 points |
| Preoperative anemia with hemoglobin ≤10 g/dL | 11 points |
| Emergency surgery | 8 points |
| Surgical incision |  |
| Upper abdominal | 15 points |
| Intrathoracic | 24 points |
| Duration of surgery |  |
| ≤2 hours | 0 points |
| 2 to 3 hours | 16 points |
| >3 hours | 23 points |

ARISCAT risk index interpretation

0 to 25 points: Low risk: 1.6% pulmonary complication rate

26 to 44 points: Intermediate risk: 13.3% pulmonary complication rate

45 to 123 points: High risk: 42.1% pulmonary complication rate
