# Supplementary material for: Prediction models for postoperative pulmonary complications in intensive care unit patients after noncardiac thoracic surgery
Source: BMC Pulm Med. 2024 Aug 29;24:420. doi: 10.1186/s12890-024-03153-z (PMC11360767; doi:10.1186/s12890-024-03153-z)
Supplement: Supplementary file 2 — Supplementary Material 2 [file 12890_2024_3153_MOESM2_ESM.docx]

Supplemental Table 1 Baseline characteristics and postoperative variables of ICU patients after non-cardiac thoracic surgery

| Variables |  | At least 1 PPC  n=171(36.00,%) | No PPC  n=304(64.00,%) | P value |
| --- | --- | --- | --- | --- |
| Age,median(IQR) |  | 61(50-68) | 57(48-68) | 0.16 |
| sex,n(%) | male | 127(75.15) | 215(70.26) |  |
|  | female | 42(24.85) | 91(29.74) | 0.26 |
| BMI,n(%) | >25 | 46(27.22) | 78(25.49) |  |
|  | <=25 | 110(65.09) | 214(69.93) | 0.53 |
| Comorbidities |  |  |  |  |
| COPD,n(%) |  | 52(30.77) | 84(27.45) | 0.44 |
| Pneumonia,n(%) |  | 20(11.83) | 32(10.46) | 0.65 |
| Hypertension,n(%) |  | 40(23.67) | 45(14.71) | 0.015* |
| Coronary heart disease(CHD),n(%) |  | 5(2.96) | 17(5.56) | 0.20 |
| Arrhythmia,n(%) |  | 7(4.14) | 7(2.29) | 0.25 |
| Diabetes,n(%) |  | 12(7.10) | 27(8.82) | 0.51 |
| Smoking,n(%) |  | 71(42.01) | 132(43.14) | 0.79 |
| Smoking index,median(IQR) |  | 500(400-800) | 600(240-800) | 0.70 |
| Drinking,n(%) |  | 58(34.32) | 108(35.29) | 0.81 |
| ASA class,n(%) | >=3 | 84(66.67) | 105(43.03) |  |
|  | <3 | 42(33.33) | 139(56.97) | 0.027* |
| ARISCAT score, median(IQR) |  | 50(50-58) | 50(43-50) | <0.001*** |
| Eurolung1(2016), median(IQR) |  | 5(3-6) | 5(3-6) | 0.13 |
| Eurolung1(2019), median(IQR) |  | 3(2.5-5) | 2.5(2.5-5) | 0.058 |
| Hemoglobin(g/L),median(IQR) |  | 130(113-143) | 133(122-144) | 0.077 |
| WBC(10^9^/L),median(IQR) |  | 6.61(5.14-9.7) | 6.135(5.01-7.99) | 0.058 |
| platelet(10^9^/L),median(IQR) |  | 181(132-224) | 183(150-241)^3^ | 0.33 |
| ALT(U/L),median(IQR) |  | 17(12-29) | 17(12-25) | 0.27 |
| AST(U/L),median(IQR) |  | 21(16-29) | 18(15-23) | <0.001*** |
| Albumin(g/L),median(IQR) |  | 40.85(35.6-44.1) | 42(39-44.7) | 0.006** |
| Urine(mmol/L),median(IQR) |  | 5.5(4.5-7) | 5.1(4.1-6.2) | 0.005** |
| creatinine(umol/L),median(IQR) |  | 78(65-90) | 74.5(63-87) | 0.21 |
| eGFR(ml/min per 1.73m^2^),median(IQR) |  | 89.695(74.34-102.82) | 91.17(80.75-101.94) | 0.35 |
| Prothrombin time(s),  median(IQR) |  | 11.1(10.5-12) | 10.85(10.3-11.4) | 0.007** |
| APTT(s),median(IQR) |  | 27.5(25.8-29.4) | 27.3(25.9-29.1) | 0.76 |
| International normalized ratio,median(IQR) |  | .99(.91-1.07) | .97(.925-1.03) | 0.038* |
| Thrombin time(s),median(IQR) |  | 17.6(16.8-18.3) | 17.7(17-18.5) | 0.13 |
| D-dimmer(ug/mL),median(IQR) |  | 1.95(.38-6.23) | .43(.195-1.11) | <0.001*** |
| 1. reactive protein(mg/L),median(IQR) |  | 11.3(3.07-81.9) | 4.51(2.89-18.25) | 0.095 |
| Procalcitonin(ng/mL)  median(IQR) |  | .24(.05-1.2) | .04(.03-.2) | 0.014* |
| Interleukin-6(pg/mL),median(IQR) |  | 6.32(2.375-85.5) | 3.685(2.36-7.7) | 0.18 |
| PaO2(mmHg),median(IQR) |  | 89.85(76.15-120.65) | 78.6(70.4-97.8) | 0.081 |
| PaCO2(mmHg),median(IQR) |  | 35.1(29.7-41.2) | 40.7(34.8-42.3) | 0.049* |
| pH,median(IQR) |  | 7.41(7.38-7.49) | 7.40(7.39-7.43) | 0.38 |
| HCO3-(mmol/L),median(IQR) |  | 23.7(19.7-23.7) | 25.15(22.2-25.6) | 0.11 |
| Lactate(mmol/L),,median(IQR) |  | 1.9(1.5-2.4) | 1.8(1.6-2.1) | 0.95 |
| Preoperative SpO2,median(IQR) |  | .98(.96-.99) | .98(.97-.99) | 0.66 |
| Pulmonary function,n(%) | none | 71(54.20) | 80(41.45) |  |
|  | normal | 21(16.03) | 50(25.91) |  |
|  | abnormal | 39(29.77) | 63(32.64) | 0.053 |
| FEV1(L),median(IQR) |  | 2.5(1.85-3.27) | 2.61(2.15-3.28) | 0.46 |
| FEV1/FVC,median(IQR) |  | .79(.71-.84) | .78(.71-.84) | 0.81 |
| FVC(L),median(IQR) |  | 3.17(2.69-3.94) | 3.315(2.79-3.93) | 0.53 |
| FEV1-ppo(L),median(IQR) |  | 1.88(1.33-2.66) | 1.87(1.39-2.27) | >0.99 |
| Echocardiography,n(%) | none | 93(55.36) | 158(52.84) |  |
|  | normal | 32(19.05) | 65(21.74) |  |
|  | abnormal | 43(25.60) | 76(25.42) | 0.84 |
| Ejection fraction,median(IQR) |  | .675(.64-71) | .68(.64-.71) | 0.37 |
| Abnormal electrocardiogram(ECG),n(%) |  | 40(38.83) | 73(34.11) | 0.019* |
| Intra-operative variables |  |  |  |  |
| Surgery type,n(%) | emergency | 46(26.90) | 32(10.60) |  |
|  | elective | 125(73.10) | 270(89.40) | <0.001*** |
| Duration of surgery,n(%) | <=2h | 18(10.65) | 57(19.32) |  |
|  | 2-3h | 22(13.02) | 43(14.58) |  |
|  | >3h | 129(76.33) | 195(66.10) | 0.033* |
| Surgical site,n(%) | lung | 26(15.20) | 67(22.19) |  |
|  | mediastinum | 57(33.33) | 90(29.80) |  |
|  | esophagus | 80(46.78) | 141(46.69) |  |
|  | Thoracic wall | 8(4.68) | 4(1.32) | 0.046* |
| Surgical approach,n(%) | VATS/RATS | 94(54.97) | 218(72.19) |  |
|  | Open chest | 71(41.52) | 76(25.17) |  |
|  | others | 6(3.51) | 8(2.65) | 0.001** |
| Blood transfusion,n(%) |  | 19(19.79) | 14(9.66) | 0.025* |
| Prophylactic antibiotics,n(%) |  | 77(80.21) | 131(90.34) | 0.056 |
| liquid(mL),median(IQR) |  | 2500(1550-3200) | 2000(1100-2700) | 0.015* |
| Variables during ICU stay, median(IQR) |  |  |  |  |
| APACHE II |  | 12(8-17) | 11(7-15) | 0.015* |
| Heart rate |  | 83.5(74.5-97.5) | 79(70-90) | <0.001*** |
| Respiratory rate |  | 15(12-18) | 14(12-17) | 0.24 |
| Systolic blood pressure(mmHg) |  | 140(120-157) | 139(124-154) | 0.94 |
| Diastolic blood pressure(mmHg) |  | 80(69-89) | 78(70-87) | 0.65 |
| Mean arterial pressure(mmHg) |  | 99(86.5-110.8) | 98.7(89-108.7) | 0.80 |
| SpO2,mean(SD) |  | 1(1-1) | 1(1-1) | 0.088 |
| FiO2,mean(SD) |  | .40(.40-.50) | .40(.40-.40) | <0.001*** |
| PaO2(mmHg) |  | 106.25(82.8-135.7) | 124(95.3-157.8) | <0.001*** |
| PaCO2(mmHg) |  | 43.1(39-47.8) | 41.8(37.2-45.5) | 0.012* |
| pH |  | 7.34(7.31-7.38) | 7.36(7.33-7.39) | <0.001*** |
| HCO3-(mmol/L) |  | 23(21-24.2) | 23.1(21.3-24.6) | 0.34 |
| Lactate(mmol/L) |  | 1.9(1.4-2.9) | 1.7(1.3-2.4) | 0.015* |
| Hemoglobin(g/L) |  | 114(98-127) | 121(109-132) | <0.001*** |
| WBC(10^9^/L) |  | 10.6(7.55-14.33) | 11.22(8.53-14.45) | 0.23 |
| Platelet(10^9^/L) |  | 147(105-211) | 164(128-209.5) | 0.043* |
| ALT(U/L) |  | 21.5(14-43) | 21(13-35) | 0.26 |
| AST(U/L) |  | 34(24-53) | 28(20-42) | <0.001*** |
| albumin(g/L) |  | 30.6(25.5-33.4) | 33.2(29.5-36.9) | <0.001*** |
| C-reactive protein(mg/L) |  | 7.84(4.41-85.9) | 4.16(2.14-8.65) | <0.001*** |
| Procalcitonin(ng/mL) |  | .175(.07-.73) | .06(.03-014) | <0.001*** |
| Interleukin-6(pg/mL) |  | 334(189-636) | 247.75(103-454.5) | 0.003** |
| Prothrombin time(s) |  | 12(11-13.5) | 11.6(10.9-12.4) | 0.002** |
| APTT(s) |  | 28.35(25.4-33.1) | 27.3(25.1-29.9) | 0.005** |
| International normalized ratio |  | 1.075(.985-1.2) | 1.04(.98-1.11) | 0.007** |
| Thrombin time(s) |  | 17.4(16.1-18.4) | 17.7(16.9-19.1) | 0.002*** |
| D-dimmer(ug/mL) |  | 3.07(1.88-4.99) | 1.89(.955-3.76) | <0.001*** |
| P/F,PaO2/FiO2 |  | 245.5(178.75-307.75) | 300.5(232.25-377) | <0.001*** |
| Ventilation mode,n(%) | A-C(VC) | 131(80.86) | 224(78.87) |  |
|  | A-C(PC) | 21(12.96) | 39(13.73) |  |
|  | SIMV(PC)+PS | 1(0.62) | 1(0.35) |  |
|  | SIMV(VC)+PS | 0(0) | 2(0.70) |  |
|  | spontaneous | 6(3.70) | 16(5.63) |  |
|  | others | 3(1.85) | 2(0.70) | 0.64 |
| Tidal volume(VT, mL) |  | 440(400-450) | 440(420-450) | 0.41 |
| PEEP(cmH2O) |  | 5(5-6) | 5(5-5) | 0.019* |
| Ventilation strategy after extubation,n(%) | Conventional oxygen | 103(60.23) | 265(87.17) |  |
|  | HFNO | 24(14.04) | 19(6.25) |  |
|  | Non-invasive | 38(23.17) | 13(4.35) |  |
|  | others | 1(0.61) | 1(0.33) | <0.001*** |
| HFNO,n(%) |  | 24(14.04) | 19(6.25) | 0.005** |
| IPAP(cmH2O) |  | 12(12-14) | 12(12-13) | 0.30 |
| EPAP(cmH2O) |  | 6(6-8) | 6(5-6) | 0.15 |
| FiO2 |  | .39(.33-.41) | .33(.33-.40) | <0.001*** |
| SpO2 |  | 1(.99-1) | 1(1-1) | 0.011* |
| PaO2(mmHg) |  | 95.15(77.3-115.4) | 106.1(85.5-142.3) | <0.001*** |
| PaCO2(mmHg) |  | 40.6(37.55-44.5) | 41.5(38.3-45.2) | 0.25 |
| pH |  | 7.39(7.36-7.44) | 7.38(7.35-7.41) | 0.023* |
| HCO3-(mmol/L) |  | 24.7(23-27.6) | 24.6(22.7-26.1) | 0.080 |
| Lactate(mmol/L) |  | 1.5(1.3-2) | 1.5(1.2-2.1) | 0.93 |
| Duration of MV,h  Median(IQR) |  | 13.8(8.18-38.33) | 8.99(3.36-14.01) | <0.001*** |
| Liquid balance,mL  Median(IQR) | pod1 | 673(112-1245) | 537.5(116-1124) | 0.22 |
|  | pod2 | 534(-218-1204.5) | 434(-130-1210) | 0.93 |
|  | pod3 | 123.55(-480-986.5) | 190(-155-854) | 0.43 |

Data were presented as mean(standard deviation,SD) or median(inter quartile range,IQR) or count and percentage;

Abbreviations: ALT,Alanine Aminotransferase;APACHE II,**Acute Physiology and Chronic Health Evaluation II**;APTT,activated partial prothrombin time;ASA,American society of Aneshesiologists;AST,aspartate transaminase;BMI,body mass index;COPD,chronic obstructive pulmonary disease;ECG,electrocardiogram;EPAP,expiratory positive airway pressure;Eurolung1, European risk models for morbidity to predict outcome following anatomic lung resections; FEV1,**Forced Expiratory Volume in one second;**FEV1-ppo,postoperative FEV1;FiO2,fraction of inspiration oxygen;FVC,forced vital capacity; HFNO,high flow nasal oxygen; ICU,intensive care unit;MV,mechanical ventilation;IPAP,inspiratory positive airway pressure;PaCO2,arterial carbon dioxide tension;PaO2,pulmonary arterial oxygen tension;PC,pressure controlled;PEEP,positive end-expiratory pressure;P/F, oxygen ratio,PaO2/FiO2;pod,postoperative days;pod,postoperative days;PPC,postoperative pulmonary complication;RATS,robot-assisted thoracic surgery; SIMV,spontaneous intermittent mandatory ventilation;SpO2,oxygen saturation;VATS,video assisted thoracic surgery;VC,volume controlled;WBC,white blood cell count.

***:p < 0.001,**:p < 0.01, *:p < 0.05.

Supplemental Table2. Baseline characteristics and outcomes between the development cohort and the validation cohort

| Variables |  | Development cohort(n=332) | Validation cohort(n=143) | P value |
| --- | --- | --- | --- | --- |
| At least 1 PPC,n(%) |  | 123(37.05) | 48(33.57) | 0.47 |
| Duration of MV(h), median(IQR) |  | 10.43(4.06-17.54) | 10.27(4.82-14.54) | 0.74 |
| Length of ICU stay(d), median(IQR) |  | 2(2-4) | 2(2-3) | 0.28 |
| LOS(d), median(IQR) |  | 11(9-16) | 11(7-14.5) | 0.056 |
| Age,median(IQR) |  | 57(48-68) | 62(52-68) | 0.041^*^ |
| sex,n(%) | male | 243(73.19) | 97(67.83) |  |
|  | female | 89(26.81) | 46(32.17) | 0.26 |
| BMI,n(%) | >25 | 90(28.57) | 34(25.56) |  |
|  | <=25 | 225(71.43) | 99(74.44)) | 0.53 |
| Comorbidities |  |  |  |  |
| COPD,n(%) |  | 98(29.52) | 46(32.17) | 0.44 |
| Pneumonia,n(%) |  | 40(12.05) | 20(13.99) | 0.65 |
| Hypertension,n(%) |  | 56(16.87) | 29(20.28) | 0.37 |
| Coronary heart disease(CHD),n(%) |  | 14(4.22) | 8(5.59) | 0.20 |
| Diabetes,n(%) |  | 23(6.93) | 16(11.19) | 0.51 |
| Smoking,n(%) |  | 131(39.46) | 53(37.06) | 0.79 |
| Smoking index(mmHg),median(IQR) |  | 500(300-800) | 600(300-800) | 0.65 |
| Drinking,n(%) |  | 117(35.24) | 49(34.27) | 0.81 |
| ASA class,n(%) | >=3 | 131(50.78) | 58(51.79) |  |
|  | <3 | 127(49.22) | 54(48.21) | 0.78 |
| ARISCAT score, median(IQR) |  | 50(47-50) | 50(47-50) | 0.15 |
| Eurolung1(2016), median(IQR) |  | 5(3-6) | 5(3-8) | 0.44 |
| Eurolung1(2019), median(IQR) |  | 2.5(2.5-5) | 3.25(2.5-5.5) | 0.37 |
| Hemoglobin(g/L),median(IQR) |  | 131.5(117-143) | 135(121-146) | 0.27 |
| WBC(10^9^/L),median(IQR) |  | 6.3(5.115-8.35) | 6.28(4.99-8.1) | 0.67 |
| Platelet(10^9^/L),median(IQR) |  | 179(138-229) | 192(152-249) | 0.063 |
| ALT(U/L),median(IQR) |  | 18(15-27) | 15(11-25) | 0.094 |
| AST(U/L),median(IQR) |  | 19(15-25) | 19(15-23) | 0.26 |
| Albumin(g/L),median(IQR) |  | 41.6(38-44.5) | 41.7(38.5-44.5) | 0.51 |
| urine(mmol/L),median(IQR) |  | 5.3(4.2-6.7) | 5(4.2-6.1) | 0.24 |
| creatinine(mmol/L),median(IQR) |  | 76(64-89) | 76(63-85) | 0.36 |
| eGFR(ml/min per 1.73m^2^),median(IQR) |  | 91(77.03-102.25) | 89.035(75.87-100.53) | 0.45 |
| Prothrombin time(s),median(IQR) |  | 10.9(10.4-11.6) | 10.9(10.25-11.8) | 0.92 |
| APTT(s),median(IQR) |  | 27.4(25.9-29.1) | 27.3(25.85-29.35) | 0.98 |
| International normalized ratio, median(IQR) |  | .98(.93-1.03) | .98(.92-1.055) | 0.81 |
| Thrombin time(s),median(IQR) |  | 17.7(17-18.4) | 17.6(16.9-18.4) | 0.42 |
| D-dimmer(ug/mL),median(IQR) |  | .56(.22-1.995) | .72(.36-3.54) | 0.11 |
| C-reactive protein(mg/L),median(IQR) |  | 6.89(3.07-34) | 6.785(2.87-57.1) | 0.84 |
| procalcitonin(ng/mL),median(IQR) |  | .06(.03-.69) | .05(.03-1.06) | 0.89 |
| Interleukin-6(pg/mL),median(IQR) |  | 4.35(2.6-12.7) | 4.62(2.22-13) | 0.89 |
| PaO2(mmHg),median(IQR) |  | 85(71.4-99.8) | 91.2(74.8-121.4) | 0.24 |
| PaCO2(mmHg),median(IQR) |  | 38.15(30.9-41.7) | 39.3(31-41.6) | 0.70 |
| pH,median(IQR) |  | 7.41(7.39-7.45) | 7.40(7.38-7.43) | 0.62 |
| HCO3-(mmol/L),median(IQR) |  | 23.5(19.7-25.6) | 23.8(21.6-25.3) | 0.54 |
| Lactate(mmol/L),median(IQR) |  | 1.8(1.5-2.13) | 1.9(1.49-3.27) | 0.55 |
| Preoperative SpO2,median(IQR) |  | .98(.97-.99) | .98(.97-.98) | 0.72 |
| Pulmonary function,n(%) | none | 108(46.96) | 43(45.74) |  |
|  | normal | 55(23.91) | 16(17.02) |  |
|  | abnormal | 67(29.13) | 35(37.23) | 0.053 |
| FEV1(L),median(IQR) |  | 2.69(2.21-3.36) | 2.35(1.62-2.76) | 0.003^**^ |
| FEV1/FVC,median(IQR) |  | .79(.72-.84) | .77(.68-.83) | 0.26 |
| FVC(L),median(IQR) |  | 3.48(2.89-4.06) | 3.06(2.55-3.47) | 0.003^**^ |
| FEV1-ppo(L),median(IQR) |  | 1.86(1.21-2.42) | 1.89(1.68-2.64) | 0.37 |
| Echocardiography,n(%) | none | 183(56.13) | 68(48.23) |  |
|  | normal | 64(19.63) | 33(23.40) |  |
|  | abnormal | 79(24.23) | 40(28.37) | 0.84 |
| Ejection fraction,median(IQR) |  | .68(.64-71) | .69(.65-.71) | 0.37 |
| Abnormal electrocardiogram(ECG),n(%) |  | 81(24.40) | 32(22.38) | 0.50 |
| Intra-operative variables |  |  |  |  |
| Surgery type,n(%) | emergency | 56(16.92) | 22(28.21) |  |
|  | elective | 275(83.08) | 56(71.79) | 0.70 |
| Duration of surgery,n(%) | <=2h | 55(17.03) | 20(14.18) |  |
|  | 2-3h | 44(13.62) | 21(14.89) |  |
|  | >3h | 224(69.35) | 100(70.92) | 0.72 |
| Surgical site,n(%) | lung | 57(17.22) | 36(25.35) |  |
|  | mediastinum | 105(31.72) | 42(29.58) |  |
|  | esophagus | 159(48.04) | 62(43.66) |  |
|  | Thoracic wall | 10(3.02) | 2(1.41) | 0.18 |
| Surgical approach,n(%) | VATS/RATS | 222(67.07) | 90(63.38) |  |
|  | Open chest | 100(30.21) | 47(33.10) |  |
|  | others | 9(2.72) | 5(3.52) | 0.72 |
| Blood transfusion,n(%) |  | 19(5.72) | 14(9.79) | 0.17 |
| Prophylactic antibiotics,n(%) |  | 138(41.57) | 55(38.46) | 0.56 |
| Liquid(mL),median(IQR) |  | 2100(1000-2800) | 2000(1400-3200) | 0.42 |
| Variables during ICU stay, median(IQR) |  |  |  |  |
| APACHE II |  | 11(7-15) | 11.5(9-17) | 0.15 |
| Heart rate |  | 81(72-94) | 79(70-90) | 0.15 |
| Respiratory rate |  | 14(12-18) | 14(12-17) | 0.80 |
| Systolic blood pressure(mmHg) |  | 140(124-156) | 137(119-153) | 0.10 |
| SpO2,median(IQR) |  | 1(1-1) | 1(1-1) | 0.10 |
| FiO2,median(IQR) |  | .40(.40-041) | .40(.40-.50) | 0.50 |
| PaO2(mmHg) |  | 118.6(88.5-154.9) | 115.75(96.55-145.1) | 0.86 |
| P/F |  | 273.55(205.75-366.5) | 278.13(220.8-341.75) | 0.93 |
| PaCO2(mmHg) |  | 42.25(37.35-46.15) | 42.3(38.7-46.15) | 0.72 |
| pH |  | 7.36(7.32-7.39) | 7.36(7.33-7.38) | 0.46 |
| HCO3-(mmol/L) |  | 23(21.2-24.7) | 23.05(21.15-24.45) | 0.45 |
| Lactate(mmol/L) |  | 1.7(1.4-2.6) | 1.9(1.4-2.4) | 0.55 |
| Hemoglobin(g/L) |  | 118(104-131) | 118(105-132) | 0.69 |
| WBC(10^9^/L) |  | 10.94(7.96-14.15) | 11.55(8.52-15.44) | 0.18 |
| Platelet(10^9^/L) |  | 154(117-207) | 166(128-213) | 0.16 |
| ALT(U/L) |  | 22(14-37) | 20(12-37) | 0.35 |
| AST(U/L) |  | 30(20-48) | 31(22-47) | 0.60 |
| albumin(pg/mL) |  | 31.9(28.25-36) | 31.7(27.3-34.9) | 0.62 |
| C-reactive protein(mg/L) |  | 7.5.7(2.51-42.35) | 6.43(3.32-25.9) | 0.52 |
| Procalcitonin(ng/mL) |  | .08(.04-.32) | .09(.04-.25) | 0.96 |
| Interleukin-6(pg/mL) |  | 275.2(119-504) | 301.15(153.5-617.35) | 0.26 |
| Prothrombin time(s) |  | 11.7(11-12.7) | 11.75(10.9-12.8) | 0.67 |
| APTT(s) |  | 27.75(25.1-31) | 27.3(25.2-30.5) | 0.79 |
| International normalized ratio |  | 1.05(.98-1.14) | 1.05(.98-1.15) | 0.65 |
| Thrombin time(s) |  | 17.7(16.7-18.8) | 17.35(16.5-19) | 0.46 |
| D-dimmer(ug/mL) |  | 2.12(1.15-4.08) | 2.29(1.26-5.1) | 0.14 |
| Ventilation mode,n(%) | A-C(VC) | 245(78.27) | 110(82.71) |  |
|  | A-C(PC) | 44(14.06) | 16(12.03) |  |
|  | SIMV(PC)+PS | 1(0.32) | 1(0.75) |  |
|  | SIMV(VC)+PS | 2(0.64) | 0 |  |
|  | spontaneous | 17(5.43) | 5(3.76) |  |
|  | others | 4(1.28) | 1(0.75) | 0.77 |
| Tidal volume(VT, mL) |  | 440(400-450) | 440(400-450) | 0.088 |
| PEEP(cmH2O) |  | 5(5-5) | 5(5-5) | 0.44 |
| Ventilation strategy after extubation,n(%) | Conventional oxygen | 257(79.32) | 111(79.86) |  |
|  | HFNO | 29(8.95) | 13(9.35) |  |
|  | Non-invasive | 36(11.11) | 1510.79) |  |
|  | others | 2(0.62) | 0 | 0.92 |
| IPAP(cmH2O) |  | 12(12-13) | 13(12-14) | 0.15 |
| EPAP(cmH2O) |  | 6(5-7) | 6(6-8) | 0.27 |
| FiO2 |  | .37(.33-.40) | .35(.33-.40) | 0.39 |
| SpO2 |  | 1(.99-1) | 1(1-1) | 0.13 |
| PaO2(mmHg) |  | 100.9(81.5-132.35) | 103.6(81-133.1) | 0.70 |
| PaCO2(mmHg) |  | 41.3(37.9-44.6) | 41.1(38.1-45.2) | 0.81 |
| pH |  | 7.39(7.36-7.42) | 7.38(7.35-7.42) | 0.24 |
| HCO3-(mmol/L) |  | 24.6(22.7-26.5) | 24.85(22.7-26.5) | 0.70 |
| Lactate(mmol/L) |  | 1.5(1.2-2) | 1.5(1.2-2.25) | 0.66 |
| Liquid balance,mL, median(IQR) | pod1 | 608.5(119.25-1166) | 530.15(66-1195) | 0.80 |
|  | pod2 | 455(-150.9-1204.5) | 467(-133.45-1201.5) | 0.94 |
|  | pod3 | 151(-415-867) | 292.7(-186.5-1062.05) | 0.14 |

Data were presented as mean(standard deviation,SD) or median(inter quartile range,IQR) or count and percentage;

Abbreviations: ALT,Alanine Aminotransferase;APACHE II,**Acute Physiology and Chronic Health Evaluation II**;APTT,activated partial prothrombin time;ASA,American society of Aneshesiologists;AST,aspartate transaminase;BMI,body mass index;COPD,chronic obstructive pulmonary disease;ECG,electrocardiogram;EPAP,expiratory positive airway pressure;Eurolung1, European risk models for morbidity to predict outcome following anatomic lung resections; FEV1,**Forced Expiratory Volume in one second;**FEV1-ppo,postoperative FEV1;FiO2,fraction of inspiration oxygen;FVC,forced vital capacity; HFNO,high flow nasal oxygen; ICU,intensive care unit;MV,mechanical ventilation;IPAP,inspiratory positive airway pressure;PaCO2,arterial carbon dioxide tension;PaO2,pulmonary arterial oxygen tension;PC,pressure controlled;PEEP,positive end-expiratory pressure;P/F, oxygen ratio,PaO2/FiO2;pod,postoperative days;pod,postoperative days;PPC,postoperative pulmonary complication;RATS,robot-assisted thoracic surgery; SIMV,spontaneous intermittent mandatory ventilation;SpO2,oxygen saturation;VATS,video assisted thoracic surgery;VC,volume controlled;WBC,white blood cell count.

***:p < 0.001,**:p < 0.01, *:p < 0.05.

Supplemental Table 3. Specific type, cumulative number of PPCs, and time of occurrence of PPCs on clinical outcomes

| Outcomes (n,%) | Type of PPCs | | | P value | Cumulative number of PPCs | | | P value | Time of occurrence of PPCs | | | P value |
| --- | --- | --- | --- | --- | --- | --- | --- | --- | --- | --- | --- | --- |
|  | Pneumonia  (153,32.21%) | Respiratory failure  (58,12.21%) | Pleural effusion  (47,9.89%) |  | 1 PPC  (94,20%) | 2 PPCs  (53,11%) | 3 PPCs  (21,5%) |  | Pod0  (49,28.65%) | Pod1  (55,32.16%) | Pod2  (34,19.88%), |  |
| Duration of MV,h  Median(IQR) | 9.90(4.00-15.78) | 11.37(5.54-17.37) | 11.7(4.54-17.95) | 0.95 | 9.90(3.73-15.81) | 11.05(6.10-17.37) | 12.5(8.17-17.37) | 0.19 | 10.24(4.54-17.37) | 11.05(4.54-17.37) | 11.20(4.54-17.33) | 0.38 |
| ICU stay,d  Median(IQR) | 2(2-3) | 2(2-6) | 2(2-6) | 0.37 | 2(2-4.5) | 3(2-4) | 2(2-2) | 0.002** | 2(2-6) | 2(2-5） | 2(1-4) | 0.94 |
| LOS,d  Median(IQR) | 11.5(8-16.5) | 12(10-19) | 12.5(10-19) | 0.045* | 12(10-20) | 13(10-19) | 14(10-18.5) | 0.20 | 13(10-19) | 12.5(10-19) | 12(10-19) | 0.069 |
| Re-intubation | 11(7.19) | 0 | 2(4.26) | 0.057 | 3(3.19) | 2(3.77) | 1(4.76) | 0.80 | 3(6.12) | 4(7.27) | 4(11.76) | 0.94 |
| Transfusion of blood or blood component | 4(2.61) | 1(1.72) | 1(2.13) | 0.076 | 4(4.26) | 4(7.55) | 1(4.76) | 0.40 | 2(4.08) | 2(3.64) | 0 | 0.86 |
| Transfusion of albumin | 17(11.11) | 8(13.79) | 5(10.64) | 0.62 | 35(37.23) | 16(30.19) | 4(19.05) | <0.001*** | 8(16.33) | 2(3.64) | 5(14.71) | 0.065 |
| Rescue during ICU stay | 11(7.19) | 2(3.45) | 3(6.38) | 0.91 | 4(4.26) | 2(3.77) | 1(4.76) | >0.99 | 3(6.12) | 3(5.45) | 5(14.71) | 0.66 |
| Re-surgery | 8(5.23) | 2(3.45) | 2(4.26) | 0.97 | 2(2.13) | 3(5.66) | 2(9.52) | 0.34 | 4(8.16) | 3(5.45) | 0 | 0.44 |
| ICU re-admission | 7(4.58) | 2(3.45) | 2(4.26) | 0.56 | 3(3.19) | 4(7.55) | 2(9.52) | 0.29 | 2(4.08) | 2(3.64) | 2(5.88) | 0.69 |
| In-hospital mortality | 1(0.65) | 0 | 1(2.13) | 0.46 | 1(1.06) | 0 | 1(4.76) | 0.43 | 1(2.04) | 0 | 0 | 0.68 |
| Automatic discharge | 8(5.23) | 2(3.45) | 1(2.13) | 0.89 | 5(3.27) | 0 | 1(4.76) | 0.33 | 3(6.12) | 3(5.45) | 2(5.88) | 0.91 |

Data were presented as count and percentage or median(inter quartile range,IQR);

Abbreviations: ICU, intensive care unit;LOS, length of hospital stay;MV,mechanical ventilation; pod, postoperative day; PPC, postoperative pulmonary complication.

***:p < 0.001,**:p < 0.01, *:p < 0.05.

Supplemental Table 4. Specific surgery procedures on clinical outcomes

| Secondary outcomes(n,%) | Surgery type | | P value | Surgery site | | | | P value | Surgery approach | | | P value | Duration of surgery | | | P value |
| --- | --- | --- | --- | --- | --- | --- | --- | --- | --- | --- | --- | --- | --- | --- | --- | --- |
|  | Emergency(78, 16.49) | Elective(395,83.51) |  | Lung(93, 19.66) | Mediastinum(147, 31.08) | Esophagus(221,46.72) | Thoracic wall(12,2.54) |  | VATS/RATS(312,65.96) | Open chest(147,31.08) | Others(14,2.96) |  | <2h(75,16.16) | 2-3h(65,14.01) | >3h(324,69.83) |  |
| PPC | 46(58.97) | 125(31.65) | <0.001*** | 26(27.96) | 57(38.78) | 80(36.20) | 8(66.67) | 0.046* | 94(30.13) | 71(48.30) | 6(42.86) | 0.001** | 18(24.00) | 22(33.85) | 129(39.81) | 0.033* |
| Duration of MV,h  (median,IQR) | 25.47(10.26-77.85) | 9.12(3.52-13.99) | <0.001*** | 10.21(3.51-14.01) | 11.88(4.26-27.54) | 9.65(4.05-13.73) | 13.47(10.26-19.48) | 0.009** | 9(3.52-15.55) | 11.56(7.66-17.06) | 8.5(2.99-107.98) | 0.0175** | 5.32(3.25-16.78) | 13.21(4.72-18.98) | 10.18(4.86-14.93) | 0.08 |
| Length of ICU stay,d  (median,IQR) | 4(2-8) | 2(2-3) | <0.001*** | 2(2-3) | 2(2-5) | 2(2-3) | 3(2-4.5) | <0.001*** | 2(2-3) | 2(2-4) | 2(2-6) | 0.0209* | 2(2-3) | 2.5(2-4) | 2(2-3) | 0.002** |
| LOS,d  (median,IQR) | 14(9-22.5) | 11(8-15) | 0.003** | 10(7-12) | 9(5-16) | 12(11-17) | 9(6-16) | <0.001*** | 11(8-15) | 12(9-18) | 14.5(10-22) | 0.0953 | 7(5-13) | 8.5(6-14) | 12(10-17) | <0.001*** |
| Re-intubation | 9(11.54) | 15(3.80) | 0.004** | 3(3.23) | 8(5.44) | 12(5.43) | 1(8.33) | 0.65 | 12(3.85) | 11(7.48) | 1(7.14) | 0.16 | 1(1.33) | 4(6.15) | 19(5.86) | 0.28 |
| Transfusion of blood or blood component | 6(7.69) | 13(3.29) | 0.070 | 4(4.30) | 7(4.76) | 7(2.17) | 1(8.33) | 0.53 | 10(3.21) | 6(4.08) | 3(21.43) | 0.021* | 3(4.00) | 3(4.62) | 13(4.01) | 0.94 |
| Transfusion of albumin | 27(34.62) | 67(16.96) | <0.001*** | 14(15.05) | 33(22.45) | 44(19.91) | 3(25.00) | 0.50 | 48(15.38) | 39(26.53) | 7(50.00) | <0.001*** | 12(16.00) | 11(16.92) | 70(21.60) | 0.44 |
| CRRT | 3(3.85) | 1(0.25) | 0.015** | 0 | 4(27.21) | 0 | 0 | 0.033* | 4(1.28) | 0 | 0 | 0.39 | 0 | 1(1.54) | 3(0.93) | 0.54 |
| Rescue during ICU stay | 6(7.69) | 17(4.30) | 0.20 | 4(4.30) | 9(6.12) | 8(3.62) | 2(16.67) | 0.17 | 12(3.85) | 10(6.80) | 1(7.14) | 0.26 | 2(2.67) | 0 | 21(6.48) | 0.049* |
| Re-surgery | 3(3.85) | 18(4.56) | 0.78 | 4(4.30) | 5(3.40) | 11(4.98) | 1(8.33) | 0.60 | 14(4.49) | 7(2.17) | 0 | >0.99 | 4(5.33) | 0 | 17(5.25) | 0.14 |
| ICU re-admission | 4(5.13) | 17(4.30) | 0.76 | 3(3.23) | 3(2.04) | 13(5.88) | 2(16.67) | 0.06 | 12(3.85) | 9(6.12) | 0 | 0.47 | 1(1.33) | 4(6.15) | 16(4.94) | 0.30 |
| In-hospital mortality | 1(1.28) | 5(1.27) | >0.99 | 0 | 3(2.04) | 3(13.57) | 0 | 0.50 | 3(0.96) | 3(2.04) | 0 | 0.49 | 1(1.33) | 1(1.54) | 4(1.23) | >0.99 |
| Automatic discharge | 11(14.10) | 7(1.77) | <0.001*** | 1(1.08) | 8(5.44) | 9(4.07) | 0 | 0.36 | 9(2.88) | 6(4.08) | 3(21.43) | 0.014* | 2(2.67) | 3(4.62) | 13(4.01) | 0.81 |

Data were presented as count and percentage or median(inter quartile range,IQR);

Abbreviations: CRRT, continuous renal replacement therapy;ICU, intensive care unit;LOS, length of hospital stay;MV,mechanical ventilation;PPC, postoperative pulmonary complication; RATS, robot-assisted thoracic surgery; VATS, video-assisted thoracic surgery.

***:p < 0.001,**:p < 0.01, *:p < 0.05.

Supplemental Table 5 Univariate and multivariate logistic regression results

| variables |  | univariate |  |  | multivariate |  |  |
| --- | --- | --- | --- | --- | --- | --- | --- |
|  |  | Odds ratio | P value | 95%CI | Odds ratio | P value | 95%CI |
| preoperative |  |  |  |  |  |  |  |
| sex |  | 1.730207 | 0.042* | 1.019879-2.935266 | 1.211841 | 0.794 | .2867064-5.122166 |
| hypertension |  | 1.905263 | 0.029* | 1.067076-3.401846 | 1.968574 | 0.296 | .5528592-7.009531 |
| ASA class>=3 |  | 2.194103 | 0.003** | 1.296754-3.712413 | 2.194103 | 0.003** | 1.296754-3.712413 |
| AST |  | 1.001129 | 0.429 | .9983346-1.00393 | 1.011148 | 0.489 | .9799066-1.043386 |
| Albumin |  | .9351925 | 0.001** | .8987111-.9731549 | .9429194 | 0.005** | .9050183-.9824077 |
| urine |  | 1.152858 | 0.003** | 1.048093-1.268096 | .9933066 | 0.970 | .6964632-1.416669 |
| creatinine |  | 1.00936 | 0.057 | .9997167-1.019096 | .985629 | 0.515 | .9435715-1.029561 |
| Lung function test |  | .8186085 | 0.207 | .5999799-1.116904 | 1.539726 | 0.445 | .5090779-4.656964 |
| FEv1-pre |  | .6062495 | 0.046** | .3708638-.9910336 | .6312011 | 0.294 | .2674465-1.489699 |
| Intra-opoerative |  |  |  |  |  |  |  |
| Surgery type |  | .6090352 | 0.001** | .4543922-.8163078 | .5625358 | 0.017** | .3503679-.9031835 |
|  | 1.emergency |  |  |  |  |  |  |
|  | 3.selective | .3220612 | 0.000*** | .1956307-.5302 | .2276739 | 0.037* | .0568533-.9117 |
| Site |  | 1.447336 | 0.013* | 1.082638-1.934886 | 2.213221 | 0.016* | 1.160756-4.21996 |
|  | 1.lung | 1 |  |  |  |  |  |
|  | 2.mediastinum | 1.177778 | 0.43 | .7876267-1.76119 | 2.37008 | 0.148 | .7362047-7.630053 |
|  | 3.esophagus | 1.003819 | 0.98 | .6892606-1.461932 | 3.736314 | 0.016* | 1.281519-10.89335 |
|  | 4.thoracic wall | 3.656441 | 0.037* | 1.084555-12.32724 | 3.46138 | 0.295 | .3389885-35.34384 |
| approach |  | 1.574537 | 0.032* | 1.04097-2.381593 | 1.503244 | 0.066 | .974033-2.319984 |
|  | 1.VATS/RATS |  |  |  |  |  |  |
|  | 2.open chest | 1.923077 | 0.008** | 1.186921-3.115814 | 1.548373 | 0.445 | .5047342-4.749942 |
|  | 3.other | 1.041667 | 0.955 | .2532741-4.28417 | 1.880369 | 0.521 | .2728573-12.95838 |
| Blood transfusion |  | .7711599 | 0.628 | .2698521-2.203754 |  |  |  |
| Liquid |  | 1.000264 | 0.063 | .9999858-1.000542 | 1.000091 | 0.634 | .9997148-1.000468 |
| during ICU stay |  |  |  |  |  |  |  |
| Heart rate |  | 1.016587 | 0.017* | 1.002934-1.030427 | 1.004457 | 0.773 | .9745995-1.035229 |
| SpO2 |  | 7.87e-11 | 0.009** | 1.95e-18-.0031729 | .1614432 | 0.920 | 5.03e-17-5.19e+14 |
| FiO2 |  | 36.98496 | 0.001** | 4.118411-332.1397 | 1185.177 | 0.134 | .1124542-1.25e+07 |
| P/F,PaO2/FiO2 |  | .9950241 | 0.000*** | .9928227-.9972303 | 1.011193 | 0.311 | .9896327-1.033222 |
| PaO2 |  | .9875097 | 0.000*** | .9813982-.9936593 | .9814046 | 0.000*** | .9718668 -.991036 |
| PaCO2 |  | 1.034603 | 0.031* | 1.003143-1.06705 | .9846874 | 0.692 | .9123986-1.062704 |
| pH |  | .0034986 | 0.005** | .0000681-.1797742 | .9592073 | 0.106 | .9120203-1.008836 |
| lactate |  | 1.077228 | 0.333 | .9265413-1.252421 | .9811414 | 0.916 | .6893467-1.396451 |
| hemoglobin |  | .9869088 | 0.022* | .97583-.9981134 | .9873735 | 0.319 | .9629997-1.012364 |
| AST |  | 1.006834 | 0.047* | 1.000076-1.013638 | 1.007748 | 0.339 | .9919399-1.023807 |
| Albumin |  | .9362586 | 0.001** | .9015326-.9723221 | .8861684 | 0.003** | .8190008-.9588446 |
| Thrombin time |  | .9962176 | 0.682 | .9782965-1.014467 | .9781466 | 0.166 | .9480416-1.009208 |
| D-dimmer |  | 1.0621 | 0.015* | 1.012001-1.11468 | .9580819 | 0.502 | .8455289-1.085617 |
| C-reactive protein |  | 1.009059 | 0.002** | 1.003318-1.014833 | 1.007649 | 0.122 | .9979626-1.017429 |
| procalcitonin |  | 1.08371 | 0.050 | .9998883-1.174559 | .9696175 | 0.601 | .8638577-1.088325 |
| interleukin-6 |  | 1.0007 | 0.095 | .999879-1.001522 | 1.000184 | 0.800 | .9987623 -1.001608 |
| Duration of MV(h) |  | 1.034985 | 0.000*** | 1.01831-1.051932 | 1.04016 | 0.000*** | 1.021001-1.059678 |
| Ventilation type after extubation |  | 1.902042 | 0.000*** | 1.519949-2.380188 | 1.016705 | 0.066 | .9989117-1.034814 |
| HFNO |  | 2.584615 | 0.013* | 1.218777-5.481097 | .6771928 | 0.658 | .1208281-3.795392 |
| FiO2 |  | 319.6611 | 0.008** | 4.417344-23132.28 | 43.55947 | 0.202 | .1326298-14306.19 |
| SpO2 |  | 1.16e-10 | 0.002** | 6.62e-17- .0002031 | 4.52e-07 | 0.215 | 4.27e-17-4798.916 |
| PaO2 |  | .9883336 | 0.002** | .9811979-.9955212 | .9929025 | 0.093 | .9846725-1.001201 |
| pH |  | 866.9246 | 0.019* | 3.051418-246298 | 3.450269 | 0.783 | .0005071-23474.42 |

Data were presented as mean(standard deviation,SD) or median(inter quartile range,IQR) or count and percentage;

Abbreviations: APACHE II,**Acute Physiology and Chronic Health Evaluation II**;APTT,activated partial prothrombin time;AST,aspartate transaminase; CI, confidence interval; ECG,electrocardiogram; Eurolung1, European risk models for morbidity to predict outcome following anatomic lung resections; FiO2,fraction of inspiration oxygen; HFNO,high flow nasal oxygen; ICU,intensive care unit;MV,mechanical ventilation;PaCO2,arterial carbon dioxide tension;PaO2,pulmonary arterial oxygen tension;PEEP,positive end-expiratory pressure;P/F, oxygen ratio,PaO2/FiO2;pod,postoperative days;PPC,postoperative pulmonary complication;RATS,robot-assisted thoracic surgery; VATS,video assisted thoracic surgery.

***:p < 0.001,**:p < 0.01, *:p < 0.05.

Supplemental Table 6. The area under curves, sensitivity and specificity of established models and reported risk models.

|  | Risk scores | AUC(95%confidence interval) | sen | spe |
| --- | --- | --- | --- | --- |
| Development cohort | Nomogram (at ICU admission) | 0.766(0.687-0.845) | 0.71 | 0.60 |
|  | Nomogram (after extubation) | 0.841(0.773-0.909) | 0.75 | 0.83 |
| Validation cohort | Nomogram (at ICU admission) | 0.703(0.503-0.903) | 0.60 | 0.79 |
|  | Nomogram (after extubation) | 0.833(0.680-0.985) | 0.79 | 0.88 |
|  | Nomogram (preoperative) | 0.711(0.656-0.766) | 0.62 | 0.73 |
| Risk scores for PPC | ASA class | 0.561(0.506-0.616) | 0.56 | 0.56 |
|  | ARISCAT score | 0.622(0.565-0.678) | 0.78 | 0.43 |
| Risk models for morbidity after thoracic surgery | Eurolung1 (2016) | 0.545(0.485-0.605) | 0.50 | 0.57 |
|  | Eurolung1 (2019) | 0.553(0.494-0.612) | 0.46 | 0.66 |
|  | STS GTSD(lung cancer) | 0.675(0.613-0.737) | 0.62 | 0.70 |
|  | STS GTSD(esophageal cancer) | 0.669(0.610-0.727) | 0.74 | 0.55 |

Abbreviations: ARISCAT, The Assess Respiratory Risk in Surgical Patients in Catalonia; ASA, American society of Aneshesiologists; AUC, area under Receiver Operating Characteristic curve; Eurolung1, European risk models for morbidity to predict outcome following anatomic lung resections; STS GTSD, The Society for Thoracic Surgeons General Thoracic Surgery Database.
